# Wild rice locus enables microbiome re-domestication for enhanced nitrogen-use efficiency

**DOI:** 10.1038/s41564-026-02498-x

**Published:** 2026-09-30

**Authors:** Jingjing Chang, Dianwen Wang, Zongmu Yao, Yingnan Pang, Hengfei Zhang, Changji Wang, Yuanbo Xie, Yu Sun, Jianfeng Zhang, Weiqiang Li, Aizhen Liang, Xiangnan Li, Dazhou Chen, Hongping Chen, Xuewen Chen, Enze Wang, Lam-Son Phan Tran, Jilin Wang, Jos M. Raaijmakers, Chunjie Tian, Eiko E. Kuramae

**Affiliations:** 1 State Key Laboratory of Black Soils Conservation and Utilization, Northeast Institute of Geography and Agroecology, Chinese Academy of Sciences, Changchun, China; 2 Department of Microbial Ecology, Netherlands Institute of Ecology NIOO-KNAW, Wageningen, the Netherlands; 3 Rice National Engineering Research Center (Nanchang), Rice Research Institute, Jiangxi Academy of Agricultural Sciences, Nanchang, China; 4 Key Laboratory of Straw Comprehensive Utilization and Black Soil Conservation, Jilin Agricultural University, Changchun, Jilin China; 5 Ecology and Biodiversity, Institute of Environmental Biology, Utrecht University, Utrecht, the Netherlands; 6 College of Landscape Architecture, Changchun University, Changchun, China; 7 Institute of Genomics for Crop Abiotic Stress Tolerance, Department of Plant and Soil Science, Texas Tech University, Lubbock, TX USA; 8 School of Agriculture and Biological Technique, Liaocheng University, Liaocheng, China

**Keywords:** Microbial ecology, Evolutionary ecology, Genetic hybridization, Bacterial host response

## Abstract

Domestication of crops, such as rice, has resulted in the loss of beneficial root-associated microbiota central to plant nitrogen-use efficiency. Whether these microbes can be inherited through hybridization of wild and cultivated plants and the host genes involved is unclear. Here we used two recombinant inbred line populations, derived from cultivated rice (*Oryza sativa*) and its wild relative *Oryza rufipogon* to map host quantitative trait loci associated with core bacterial taxa. Integrating microbiome profiling, quantitative trait loci mapping, population genomics and transgenic validation, we identified 113 candidate plant genes. The receptor-like kinase-encoding gene *OsRLK*, found predominantly in wild rice, was significantly associated with *Acinetobacter*, which enhances plant nitrogen uptake. Knockout and overexpression of *OsRLK*, inoculated with an *Acinetobacter* synthetic community, validated their role in nitrogen-use efficiency. In field trials, the overexpression line significantly increased yield-related traits. These findings establish the genetic basis of microbiome re-domestication and provide a framework for microbiome-guided breeding of nutrient-efficient crops.

## Abstract (web summary)

Hybridization of wild and cultivated rice plants improves nitrogen use efficiency through microbiome re-domestication.

## Main

Ensuring sustainable food production while reducing environmental impacts is a major challenge^1^. Intensive fertilizer and pesticide use has increased crop yields but also caused environmental degradation^2–4^. Sustainable strategies are therefore needed to improve crop nutrient-use efficiency (NUE) and yield. Enriching beneficial plant-associated microbes can enhance nutrient uptake and stress resilience, reducing reliance on synthetic fertilizers and pesticides^5,6^.

Extending the microbiome rewilding hypothesis^7^, which proposes restoring microbial partners lost during domestication, we asked whether this process is genetically controlled by host loci. We hypothesized that beneficial microbiota and their functions can be restored in cultivated crops through the inheritance of host genetic loci from wild relatives, a process we term ‘microbiome re-domestication’. We therefore analysed wild rice microbiomes to identify key ‘ancestral’ microbiota and corresponding microbiome-associated genes (*M*-genes), defined as host genes involved in microbiome recruitment and function that were counterselected during domestication and breeding.

Modern varieties were primarily bred for above-ground agronomic traits under high-input conditions, potentially overlooking beneficial below-ground microbiota adapted to low-input or stress environments. Growing evidence shows that microbiome selection differs between wild accessions and cultivated plants^8–10^. Studies using tomato (*Solanum lycopersicum*) and barley (*Hordeum vulgare*) recombinant inbred line (RIL) populations demonstrated associations between plant loci and rhizosphere microbiome composition^11–13^. Despite these advances, previous studies have focused on statistical associations between host loci and microbiome composition under controlled conditions, without addressing whether beneficial microbiome member(s) from wild relatives can be stably reinstated in crop progeny or contribute to agronomic traits under real field conditions. In particular, it remains unclear whether core microbiome members can be retained following hybridization and genetic recombination in RIL populations, and whether specific host loci can restore the functions of these microbes associated with NUE.

We previously showed that wild rice (*Oryza rufipogon*) hosts a core rhizobacterial community that promotes nutrient acquisition under nutrient limitation and provides a valuable resource for *M-*gene-based crop improvement^14^. We further demonstrated that wild-rice rhizosphere exhibits higher abundance and activity of nitrogen fixation gene, whereas cultivated rice is enriched in genes for N_2_O production and emissions^15^. This differentiation is partially reproduced by root exudates^15^, linking wild rice genetics and root chemistry to microbial recruitment supporting nitrogen acquisition and reduced greenhouse gas emissions.

**Fig. 1:**
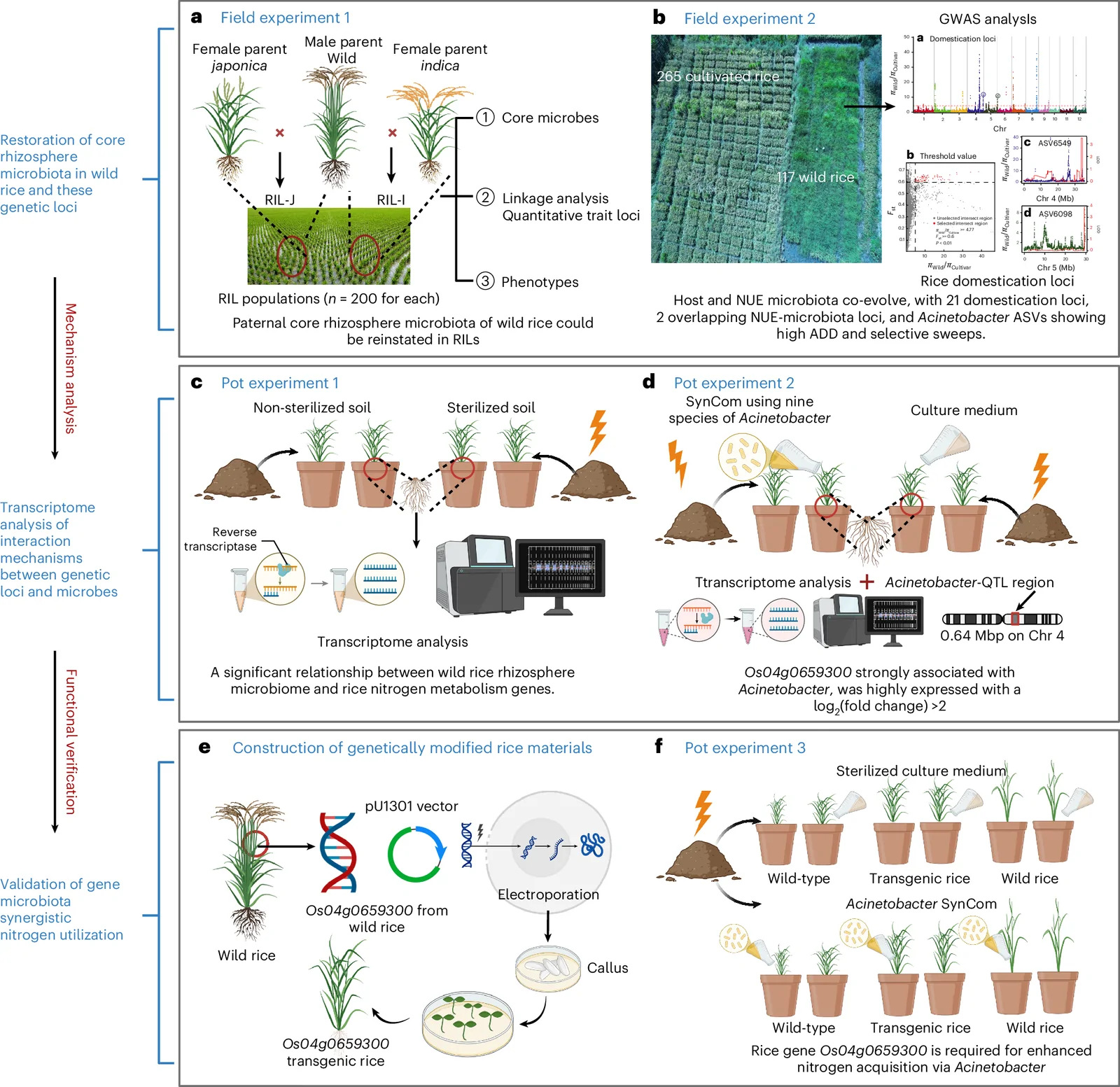
A three-phase experimental framework linking rice domestication loci, rhizosphere microbiota and nitrogen-use efficiency (NUE). **a**, Field experiment 1 to identify the core bacterial groups in the rhizosphere of wild rice and linkage analysis. **b**, Field experiment 2 to examine the overlap between core bacterial taxa-related QTLs and domestication loci. **c**, Pot experiment 1 using transcriptomic analysis to reveal the impact of wild-rice rhizosphere microbiome on plants. **d**, Pot experiment 2 using transcriptomic analysis to investigate the impact of *Acinetobacter* on plants. **e**, Construction of genetically modified rice material. **f**, Pot experiment 3 to validate the genetically modified rice materials in regulating *Acinetobacter*. LOD, logarithm of odds. Created in BioRender: Chang, J. https://BioRender.com/zl2dod2 (2026).

In this study, we investigated whether beneficial rhizosphere microbiota lost during rice domestication can be genetically recovered in hybrid progeny through the introgression of wild-rice loci. We first assessed the rhizosphere microbiota assembly in two RIL populations derived from wild rice (*O. rufipogon*) and cultivated rice (*O. sativa* L. ssp. *japonica* and *indica*) and determined whether paternal wild-rice core microbiota could be reinstated in RILs grown under field conditions (Fig. 1). We then mapped quantitative trait loci (QTLs) associated with core taxa and overlapped microbiome-associated QTLs with domestication loci identified from whole-genome data of 382 rice accessions. Subsequent transcriptome analyses and microbiome profiling identified candidate genes, particularly the receptor-like kinase-encoding gene *OsRLK* (*Os04g0659300*), whose association with an *Acinetobacter* synthetic community (SynCom) was functionally validated using overexpression and knockout rice lines.

Collectively, by integrating genetics, genomics and rhizosphere microbiome profiling, we link *OsRLK* to the reinstatement of wild-rice-derived bacteria that improve nitrogen-related functions and support enhanced plant performance in domesticated rice. This study goes beyond previous association-based studies by connecting host genetic loci to functionally relevant core microbiota and nitrogen-use efficiency under field conditions.

## Results

### Rhizobacterial diversity and core microbiome identification

**Extended Data Fig. 1:**
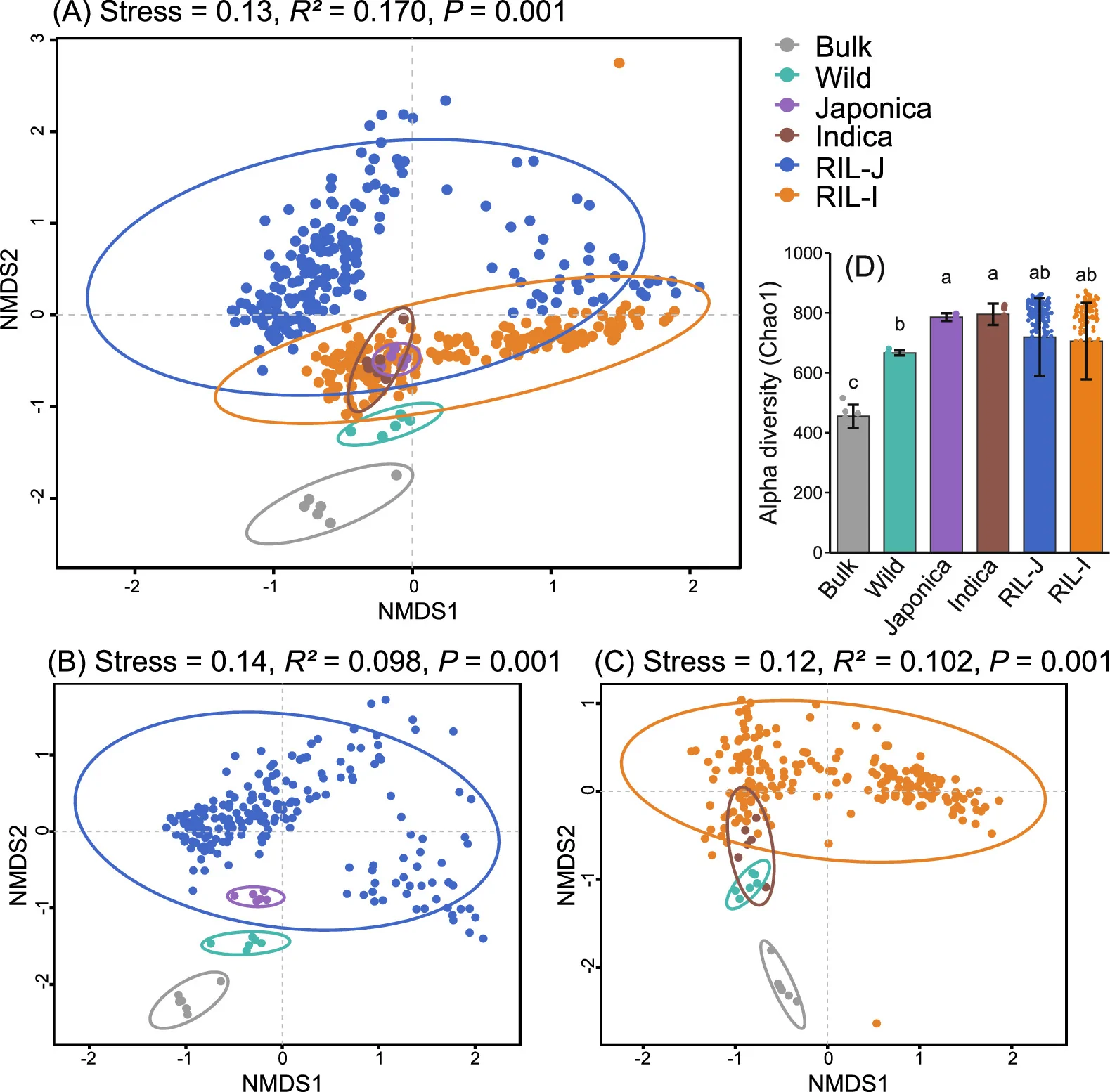
Non-metric multidimensional scaling (NMDS) and alpha diversity of rhizosphere bacterial community in the parental lines and recombinant inbred line (RIL) populations. (**A**) All the samples, (**B**) Wild rice, *japonica* and RIL-*japonica* (RIL-J), (**C**) Wild rice, *indica* and RIL-*indica* (RIL-I). NMDS analysis is based on the Bray-Curtis dissimilarity matrix at the amplicon sequence variant (ASV) level. 95 % confidence ellipses are colored as follow: orange for wild rice, purple for *japonica* rice, brown for *indica* rice, blue for bulk soil, green for RIL-J, and red for RIL-I. (**D**) Alpha diversity of rhizosphere bacterial community in all the samples (one-way ANOVA, *P* < 0.05). Source data

To characterize the core rhizosphere bacteria of paternal wild rice, we performed 16S ribosomal (r)RNA amplicon sequencing on 424 samples, including bulk soil (*n* = 6), parental rhizosphere samples (*n* = 6 per genotype) and 2 RIL populations (RIL-J and RIL-I), each comprising 100 lines and 200 rhizosphere samples in total (2 replicates per line). Rhizosphere communities differed in alpha diversity (Chao1 index) and composition from bulk soil (Extended Data Fig. 1, permutational multivariate analysis of variance (PERMANOVA), *R*^2^ = 0.17, *P* = 0.001).

**Extended Data Fig. 2:**
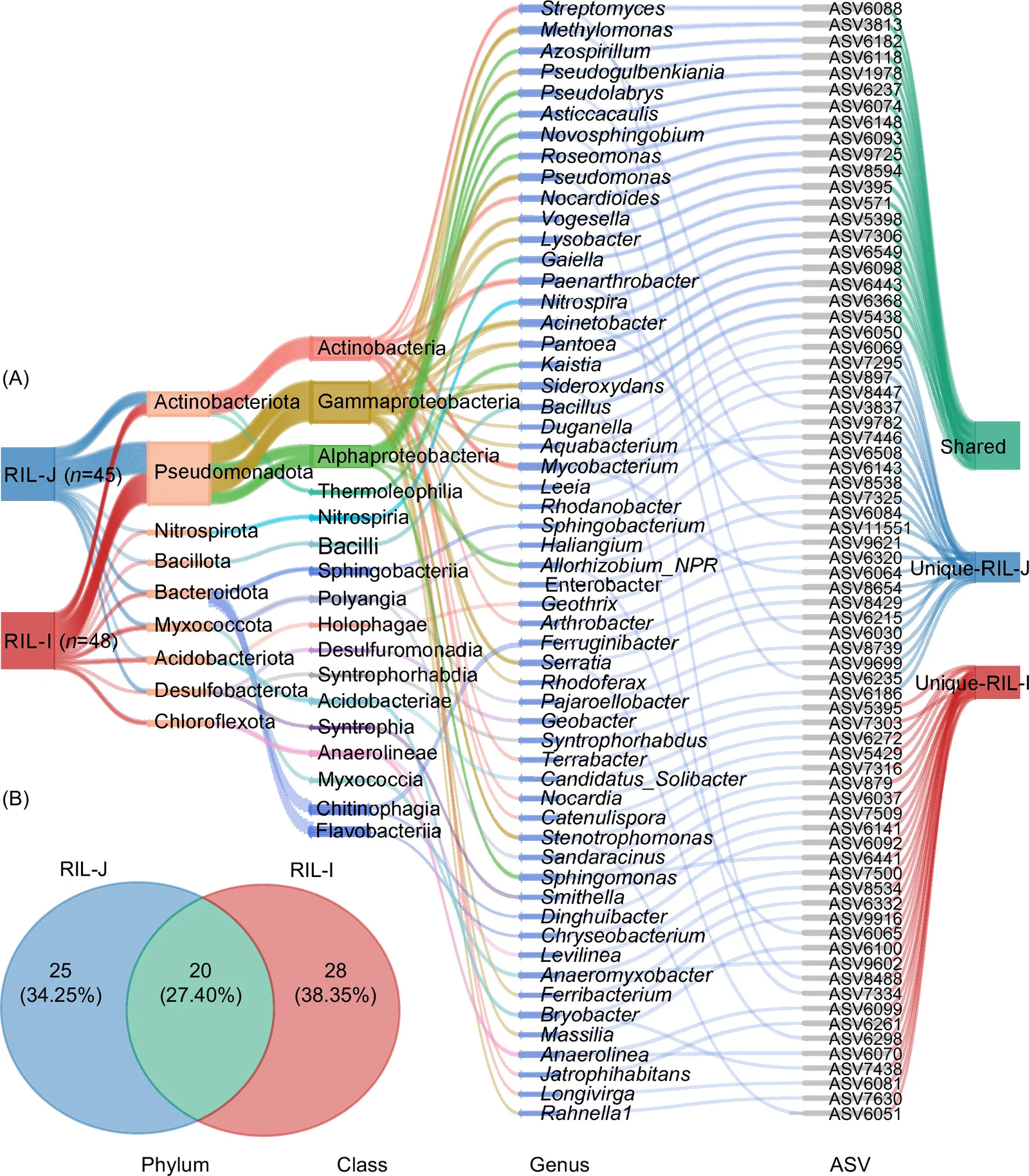
Core rhizosphere bacterial community regulated by paternal wild rice only in two recombinant inbred line (RIL) populations. (**A**) Visualization of the paternal wild rice-specific core rhizosphere community transferred to RIL-*japonica* (RIL-J) and RIL-*indica* (RIL-I) populations by excluding the core bacterial taxa shared with maternal *japonica* and *indica* rice, respectively. (**B**) Pie charts illustrating the numbers of shared and unique amplicon sequence variants (ASVs) between the RIL-J and RIL-I core rhizosphere microbes. Source data

To define the paternal wild-rice-specific core rhizosphere bacteria, we first identified core bacterial taxa in the wild rice, *japonica* and *indica* parental lines using generalized joint attribute modelling (GJAM) analyses and then excluded core bacterial taxa shared with the maternal *japonica* or *indica* parents. The remaining bacterial taxa were defined as paternal wild-rice-specific core members and tracked in the RIL populations (Extended Data Fig. 2). The RIL-J core comprised 45 amplicon sequence variants (ASVs), while the RIL-I core comprised 48 ASVs (Extended Data Fig. 2a). Of these, 27.40% (*n* = 20) of ASVs were shared, while 34.25% (25) and 38.35% (*n* = 28) were unique to RIL-J and RIL-I, respectively (Extended Data Fig. 2b).

**Fig. 2:**
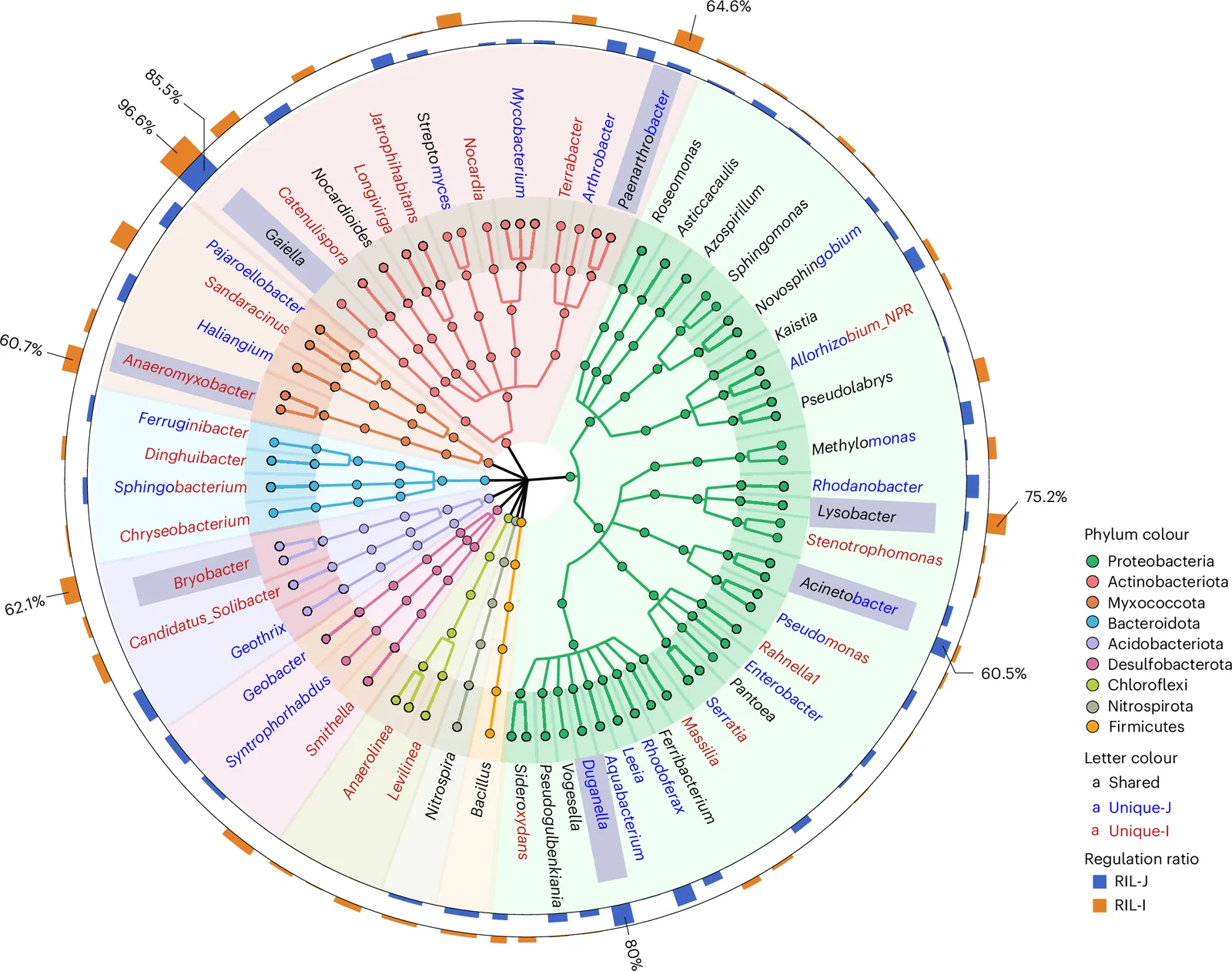
Percentage of core bacterial taxon in two RIL populations. The two outer rings display coloured circles representing the percentage of RIL accessions in which each core taxon was detected across the RIL-*japonica* (RIL-J) and RIL-*indica* (RIL-I) populations (*n* = 200 per population). The regulation ratio of the core rhizosphere bacterial community influenced by paternal wild rice in the two RIL populations was calculated as the number of RIL accessions containing the taxa divided by the total number of RIL accessions. Source data

The 20 core bacterial taxa (ASVs) were assigned to *Acinetobacter*, *Asticcacaulis*, *Azospirillum*, *Bacillus*, *Gaiella*, *Kaistia*, *Lysobacter*, *Methylomonas*, *Nitrospira*, *Nocardioides*, *Novosphingobium*, *Paenarthrobacter*, *Pantoea*, *Pseudogulbenkiania*, *Pseudolabrys*, *Pseudomonas*, *Roseomonas*, *Sideroxydans*, *Streptomyces* and *Vogesella*, and were present in different frequencies within the 2 RIL populations relative to the paternal wild rice (Fig. 2). To assess the influence of paternal wild rice on the 2 RIL populations, we calculated the percentage of each core taxon across 200 RIL accessions. *Gaiella* occurred in 88.5% of RIL-J accessions and 96.6% of RIL-I accessions (Fig. 2). *Acinetobacter*, *Paenarthrobacter* and *Duganella* reached 60.5%, 64.6% and 80%, respectively, in RIL-J while *Bryobacter*, *Lysobacter*, *Anaeromyxobacter*, reached 62%, 75.2% and 60.7%, respectively, in RIL-I, demonstrating that paternal core bacterial taxa are maintained and detectable in the RIL progeny.

### Associations between core bacterial taxa and rice phenotypes

**Extended Data Fig. 3:**
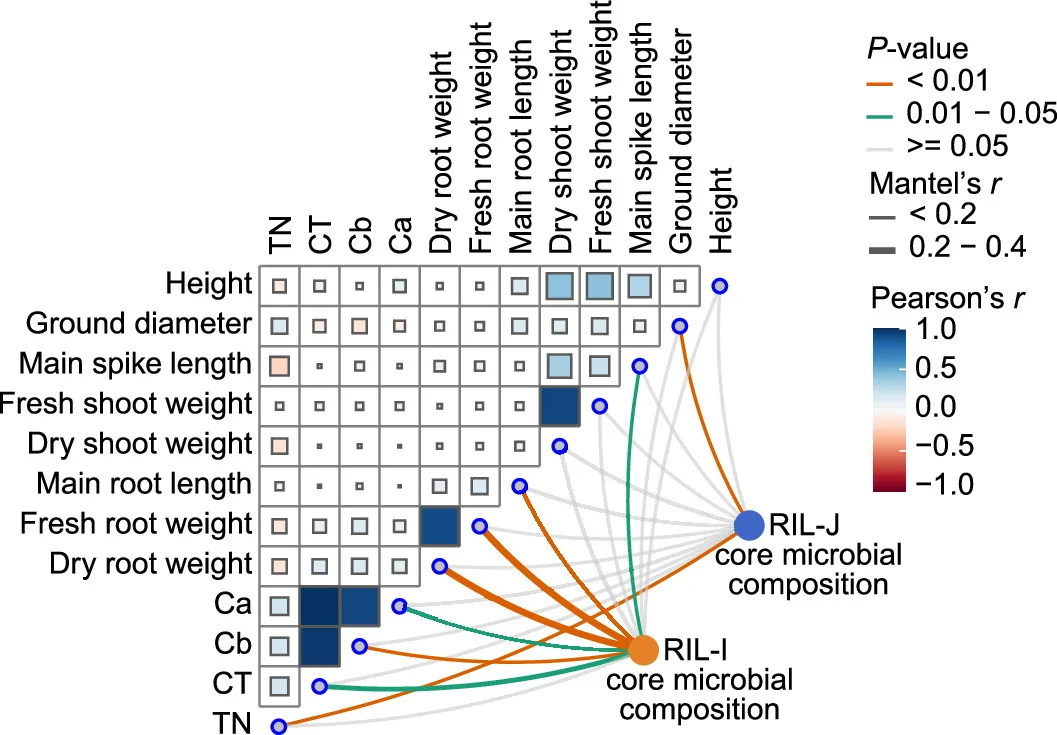
Relationships between core rhizosphere bacterial community and plant physiological and biochemical properties. This figure depicts the paternal wild rice-specific core bacteria community in the RIL-*japonica* (RIL-J) and RIL-*indica* (RIL-I) populations, derived by excluding bacterial taxa shared with *japonica* and *indica* rice. Pairwise comparisons of plant physiological and biochemical properties are visualized using a color gradient based on Pearson’s correlation coefficients. In this matrix, blue squares (gradient from light to dark) indicate positive correlations (0 < r ≤ 1), red squares (gradient from light to dark) indicate negative correlations (-1 ≤ r < 0), and color intensity scales with the absolute value of the correlation coefficient (|r|). The relationship between core bacterial community composition and each environmental factor was assessed by using a Mantel test. Edge width corresponds to Mantel’s r statistic, indicating the strength of distance correlations, while edge color represents statistical significance based on 9,999 permutations. TN = total nitrogen, Ca = chlorophyll a, Cb = chlorophyll b, CT = total chlorophylls. Source data

Partial Mantel tests were performed to assess correlations between the abundances of the core bacterial taxa and the phenotypes of the two RIL populations of rice, including plant height, fresh and dry shoot weights, fresh and dry root weights, main spike length, ground diameter, chlorophyll *a* (*Ca*), chlorophyll *b* (*Cb*), total chlorophyll (*C*T) and total nitrogen (TN) content (Extended Data Fig. 3).

In RIL-J, plant ground diameter and TN significantly correlated with the core bacterial taxa, while shoot and root biomass and CT did not (Extended Data Fig. 3). Conversely, in RIL-I, fresh and dry root weights, *Ca*, main spike length, *C*T and *Cb* significantly correlated with the core bacterial taxa (Extended Data Fig. 3).

### Genetic loci associated with core bacterial taxa in rice

**Fig. 3:**
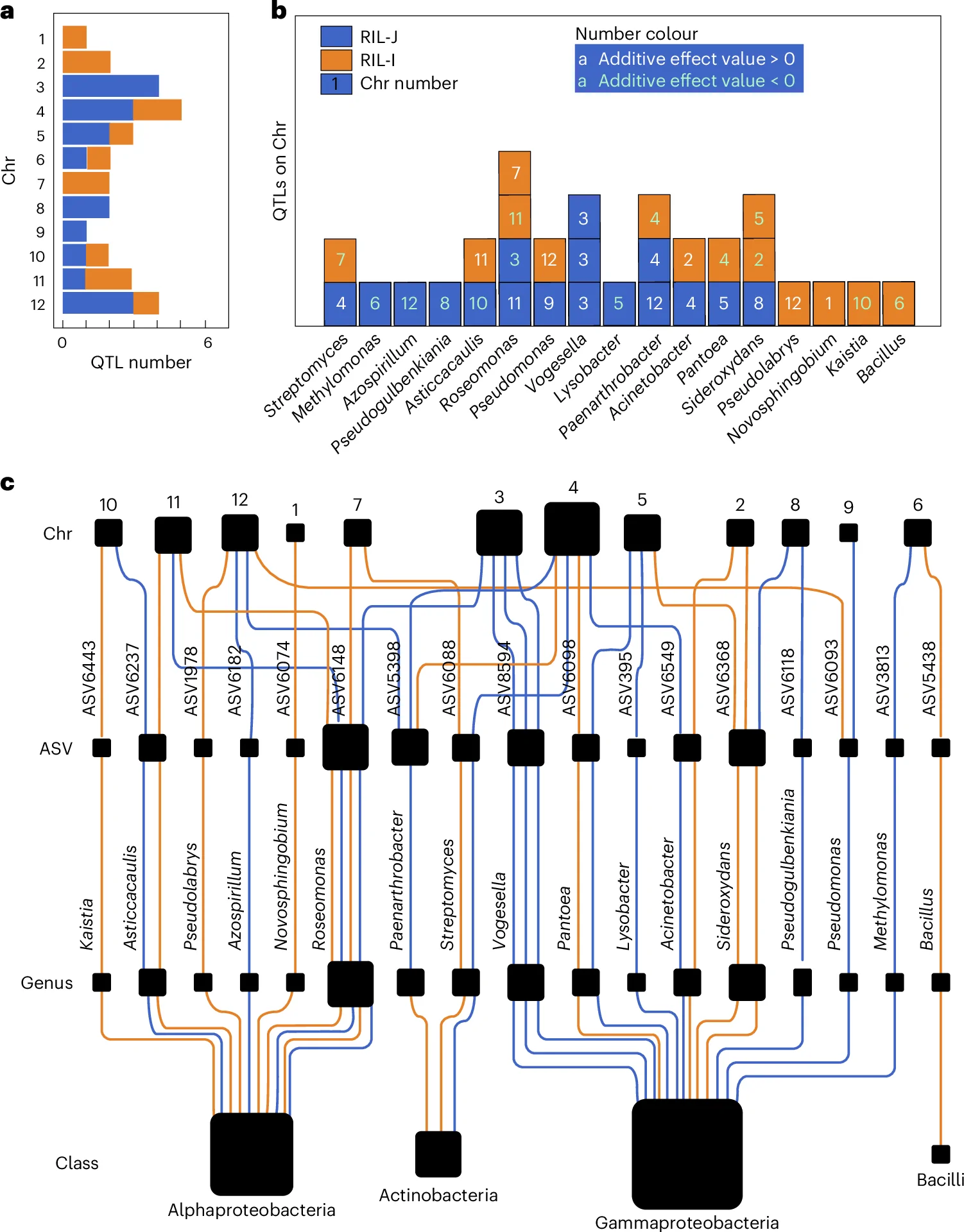
QTLs in RILs associated with the core ASVs common to wild, RIL-J and RIL-I populations transferred from the paternal wild rice. **a**, Colour-coded summary of the number of core bacterial ASVs–QTLs identified per chromosome (Chr) in the 2 RIL populations. **b**, Summary of the core ASVs by genus, with the chromosome number of each associated QTL indicated within each square. The *x* axis represents bacterial genera, whereas the *y* axis represents the number of chromosomes associated with these bacterial genera. **c**, Hierarchical network illustrating core bacterial ASVs–QTLs. The top row of nodes represents rice chromosomes, linked to specific core ASVs below them. These ASVs connect to various genera and bacterial classes. Node sizes for chromosomes reflect the number of outbound edges, and sizes for ASVs, genera and classes are based on inbound edges. QTL mapping was performed using *n* = 76 *O. sativa L*. ssp. *japonica* RILs and *n* = 90 *O. sativa L*. ssp. *indica* RILs. Source data

Linkage analysis of the 20 core ASVs common to wild, RIL-J and RIL-I populations identified 31 QTL peaks (logarithm of odds (LOD) > 2) associated with the abundance of 17 core ASVs. In RIL-J, 17 QTLs were associated with 13 core ASVs, while in RIL-I, 14 QTLs were linked to 12 core ASVs (Fig. 3 and Supplementary Table 1). Using wild-rice allele as reference, negative effects were relatively more associated with the cultivated rice allele and positive effects with the wild-rice allele. Of the lineages with multiple QTLs, *Acinetobacter* and *Vogesella* were wild allele enriched (Fig. 3b). The chromosomal locations associated with each core bacterial taxon differed between RIL-J and RIL-I. For example, *Acinetobacter* was linked to a QTL on chromosome (Chr) 4 in RIL-J but to a QTL on Chr 2 in RIL-I, while *Sideroxydans* was associated with a QTL on Chr 8 in RIL-J but with 2 QTLs on Chr 2 and 5 in RIL-I (Fig. 3c). Together, these analyses indicate that wild-rice loci consistently influence the abundance of specific core bacterial taxa, with *Acinetobacter* representing one of the microbial taxa across populations.

### Rice NUE genes linked to core bacterial functions

**Extended Data Fig. 4:**
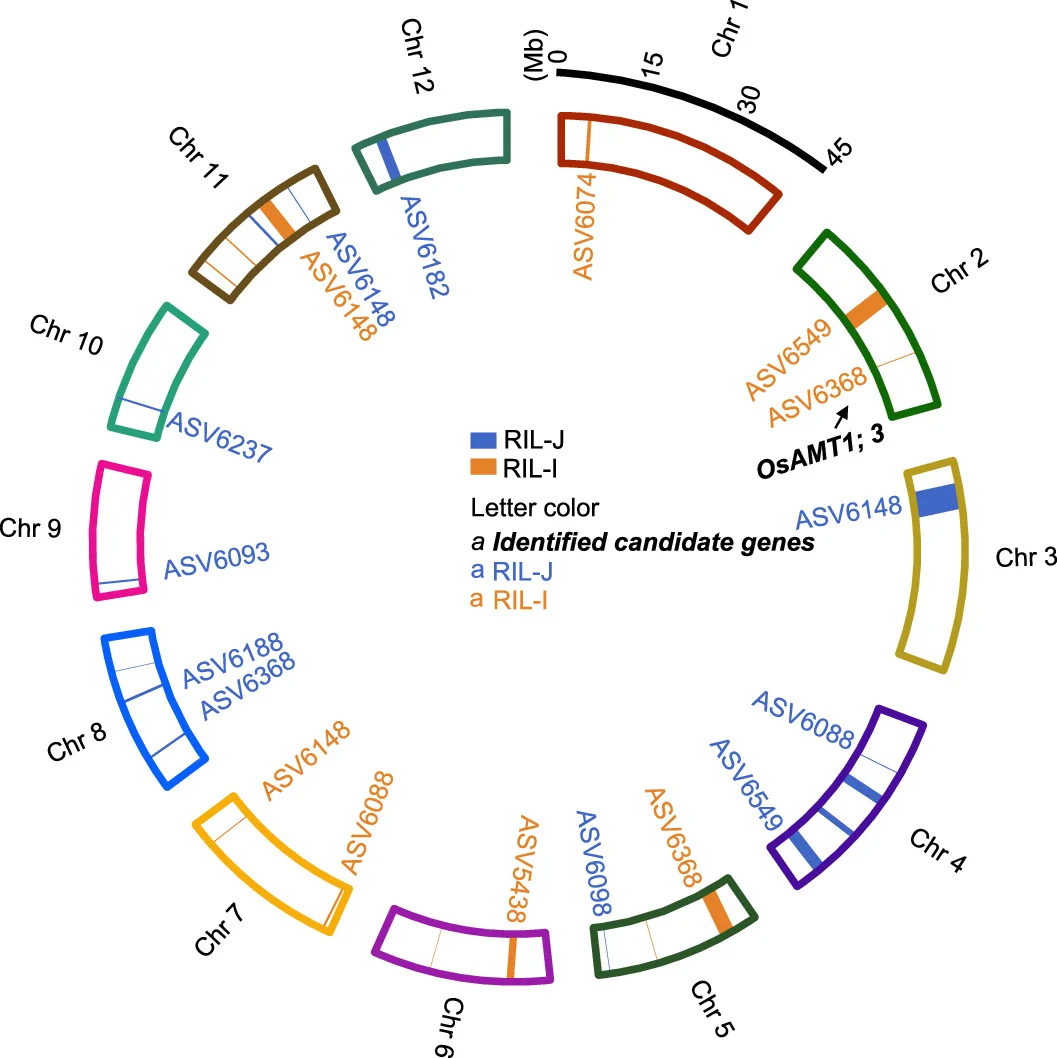
Quantitative trait locus (QTL) mapping intervals of the core ASVs common to wild, RIL-J and RIL-I populations related to nitrogen-use-efficiency (NUE) and NUE-associated rice candidate genes identified by transcriptome analyses. The 12 consensus rice chromosomes are depicted as 12 circular bars. Ticks on the chromosomes indicate the marker positions. Identified NUE-associated candidate rice genes coinciding with NUE-related core bacterial taxa are shown in italics. See Supplementary Table 2 for detailed supporting references. Source data

Given the urgent need to improve NUE in rice systems, where only ~30–40% of applied nitrogen is utilized by plants, we focused on microbial functions related to nutrient cycling. Previous studies showed that nitrogen-cycling-related microbes are enriched in wild rice^14,15^; however, their genetic basis in host control remains unclear. Using FAPROTAX^16^ for mapping taxonomy to validated functions, we inferred functions of the 20 core ASVs shared by wild, RIL-J and RIL-I. Ten ASVs in nitrogen metabolism were associated with genes on Chr 3, 4, 5, 8, 9, 10 in RIL-J and Chr 1, 2 and 6 in RIL-I (Extended Data Fig. 4). Seven ASVs including *Acinetobacter* (ASV6549), *Novosphingobium* (ASV6074), *Sideroxydans* (ASV6368), *Streptomyces* (ASV6088), *Pantoea* (ASV6098), *Bacillus* (ASV5438) and *Pseudomonas* (ASV6093) were predicted to be nitrogen fixers (Supplementary Table 2).

We identified 10 ASVs predicted to be involved in nitrogen metabolism. Among them, one ASV was associated with the previously reported rice NUE gene *OsAMT1;3*, which encodes an ammonium transporter^17^. Specifically, the gene was associated with *Sideroxydans* (ASV6368) in RIL-I (Supplementary Table 2). Notably, the remaining ASVs were linked to unreported NUE-related rice genes. For instance, 113 rice genes, including *OsRLK* (*Os04g0659300*) were significantly associated with *Acinetobacter* (ASV6549) abundance (Supplementary Table 3).

### QTLs associated with core bacterial taxa overlap rice domestication loci

**Fig. 4:**
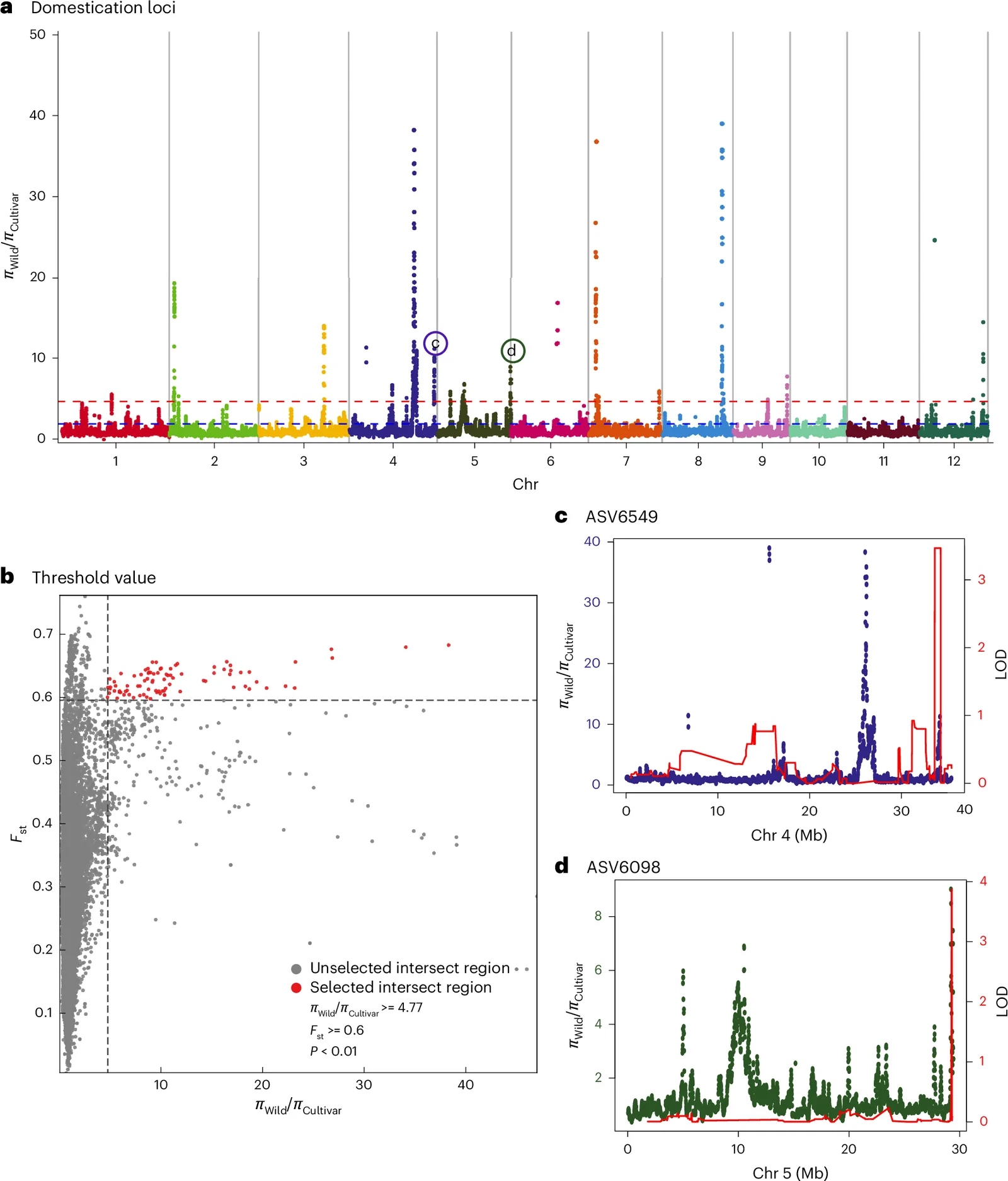
Whole-genome screening shows that domestication loci coincide with rice loci related to NUE-associated core bacterial taxa. **a**, Whole-genome screening of domestication loci in *Oryza rufipogon* and *O. sativa* using genomic variation data. The *π*_wild_/*π*_cultivar_ values are plotted against each chromosomal position, with horizontal dashed lines representing genome-wide selection signal thresholds at top 1% (*π*_wild_/*π*_cultivar_ > 4.77, red line) and top 5% (*π*_wild_/*π*_cultivar_ > 1.99, blue line). **b**, Population fixation statistics (*F*_st_) and *π*_wild_/*π*_cultivar_ values for the whole-genome screening. A top 1% threshold identifies highly differentiated regions. **c**,**d**, Strong selective sweeps in domestication loci associated with NUE-related core bacterial ASV6549 (**c**) and ASV6098 (**d**) on chromosomes 4 and 5, respectively. Logarithm of odds (LOD) values from composite interval mapping are plotted against chromosomal positions. Source data

**Extended Data Fig. 5:**
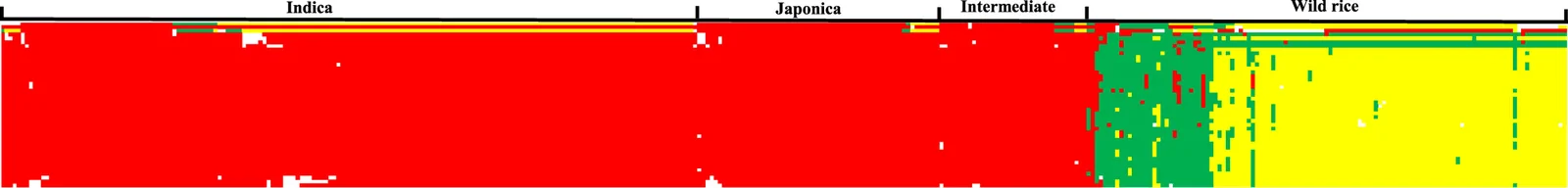
Natural variation in *OsRLK* gene among 377 rice accessions compared to the Nipponbare (MSU7) reference genome. Natural variation in *OsRLK* gene among 382 rice accessions compared to the Nipponbare (MSU7) reference genome. Color coding: red for nucleotide sequences identical to the reference genotype, yellow for differences from the reference, blue-green for heterozygosity, and white for missing sequences. Includes cultivated rice accessions from *japonica*, *indica*, and intermediate groups. Source data

To explore whether the host and rhizosphere core bacterial taxa co-evolved, we sequenced the whole genomes of 382 rice accessions, including 117 wild-rice *O. rufipogon* accessions and 265 cultivated rice *O. sativa* accessions (Extended Data Fig. 5 and Supplementary Table 4). We estimate genetic diversity ratio (*π*_wild_/*π*_cultivar_) between wild and cultivated rice accessions and identify rice domestication loci (Fig. 4a,b). This analysis identified 21 domestication loci across all chromosomes except Chr 10 and 11 (Supplementary Table 5). Comparing QTLs for the 20 core ASVs shared by wild, RIL-J and RIL-I populations with these loci revealed two strong selective sweeps overlapping with QTLs associated with *Acinetobacter* ASV6549 and *Pantoea* ASV6098 (Fig. 4c,d). The locus associated with *Acinetobacter* showed higher additive effect value (ADD), phenotypic variance explained (PVE), along with stronger selective sweep signals (Supplementary Table 2), and was therefore selected for isolation and subsequent bioassays to assess their potential contribution to nitrogen acquisition.

### Rice candidate genes associated with *Acinetobacter*

**Extended Data Fig. 6:**
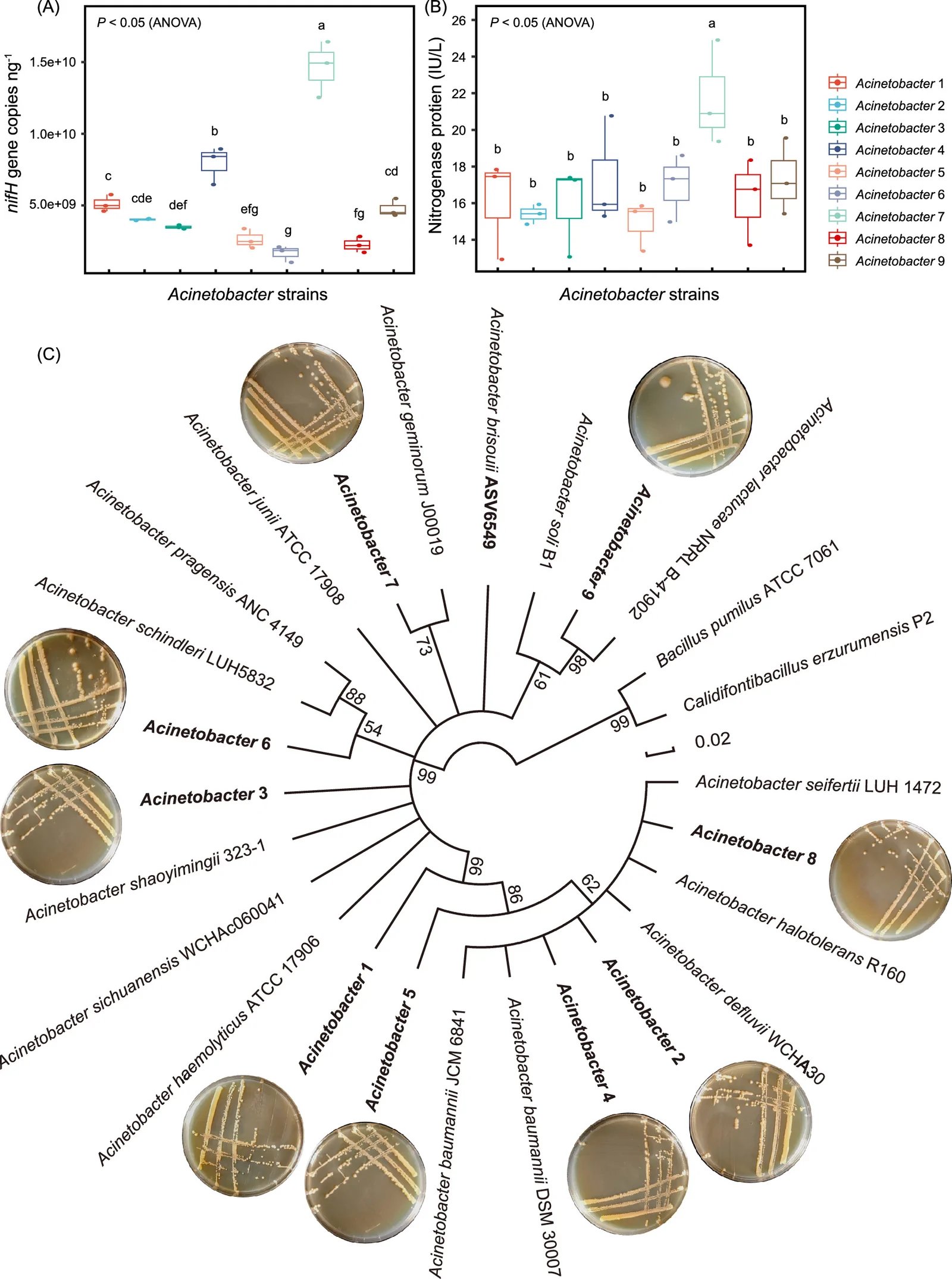
Functional validation and phylogenetic characterization of *Acinetobacter* isolates used in this study. (**A**) Quantification of the *nifH* gene in nine *Acinetobacter* isolates by qPCR. (**B**) Nitrogenase protein abundance of the isolates measured under pure culture conditions. T-test, *P* < 0.05, n = 3. (**C**) Phylogenetic relationships and images of the *Acinetobacter* isolates based on 16S rRNA gene sequences. Source data

Nine *Acinetobacter* strains exhibiting high 16S rRNA gene similarity to *A. baumannii*, *A. halotolerans*, *A. sichuanensis*, *A. schindleri, A. geminorum*, *A. defluvii* and *A. lactucae* were isolated from wild-rice rhizosphere soil to make a SynCom (Extended Data Fig. 6c). Notably, *nifH* and nitrogenase protein abundance in *Acinetobacter* 7 was significantly higher than in the other 8 *Acinetobacter* strains, indicating potential functional heterogeneity in nitrogen-related traits among the strains (Extended Data Fig. 6a,b).

**Fig. 5:**
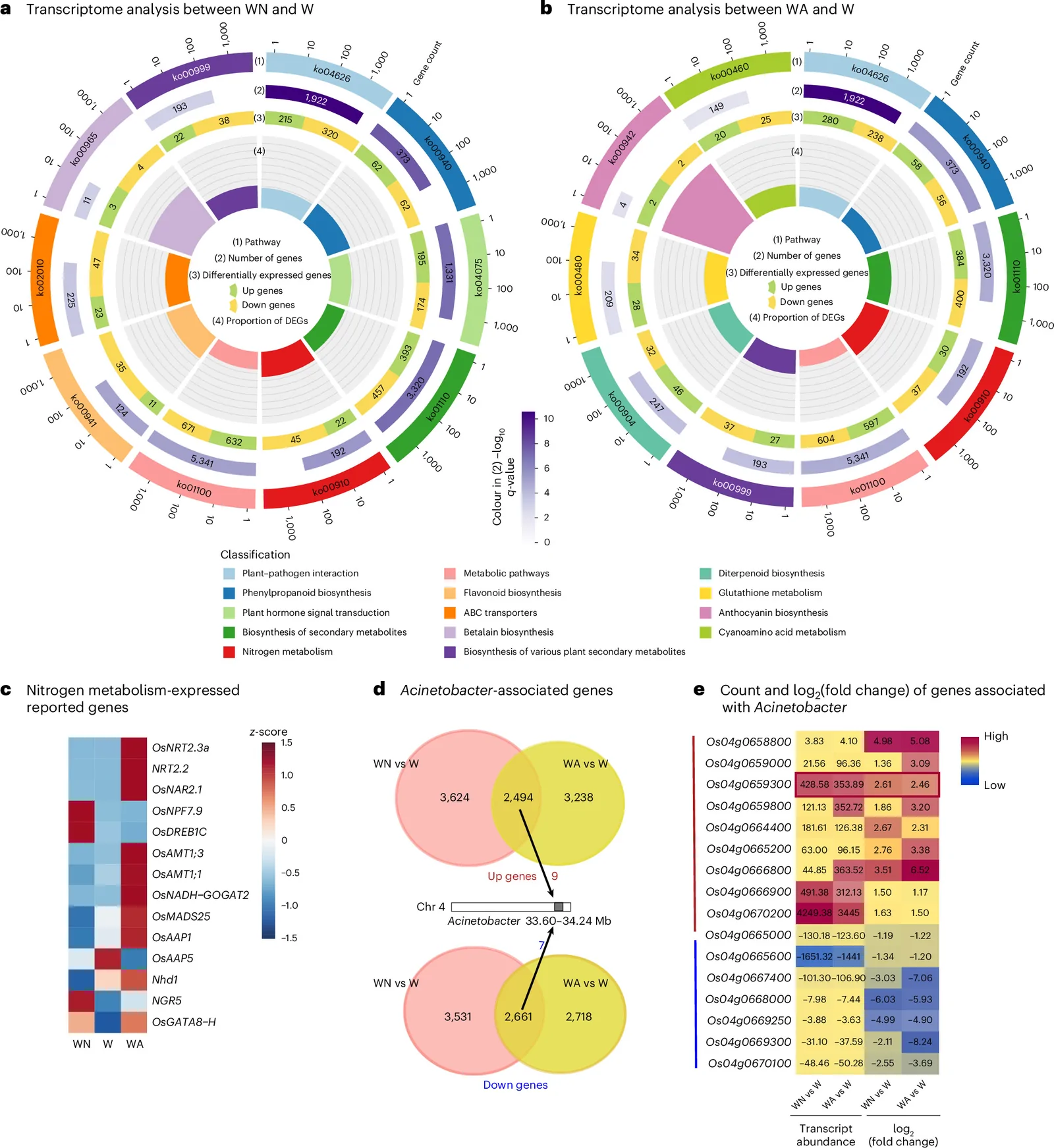
Root transcriptome analyses of wild rice grown in non-sterilized soil (WN) vs sterilized soil (W), and sterilized soil inoculated with the synthetic *Acinetobacter* community (WA) vs sterilized soil. **a**, KEGG pathway enrichment analysis showing highly significant (Benjamini–Hochberg method, *P* < 0.01) pathways in WN vs W. **b**, KEGG pathway enrichment analysis showing highly significant (Benjamini–Hochberg method, *P* < 0.01) pathways in WA vs W. The first layer displays the top 10 KEGG pathways, with the outermost scale indicating the number of genes in each pathway on a logarithmic scale (gene count; no unit). The second layer shows the number of genes in the genomic background and *q*-values for gene enrichment in these pathways, with colour indicating the −log_10_ transformed *q*-value for pathway enrichment. The third layer presents a bar graph of the number of enriched genes. The fourth layer shows the proportion of enriched genes within all genes involved in each KEGG pathway. **c**, Expressed reported genes involved in nitrogen metabolism. Expression values of 14 reported N metabolism genes were *z*-score normalized and visualized on a unified color scale ranging from −1.5 (blue, low) to 1.5 (red, high), with white indicating the mid point. **d**, Venn diagram showing upregulated and downregulated genes in wild rice under different conditions: W (sterilized soil), WN (non-sterilized soil) and WA (sterilized soil with *Acinetobacter* synthetic community). The *Acinetobacter*-associated 0.64 Mbp region includes 7 upregulated and 3 overlapping downregulated overlapping genes. **e**, Count and log_2_(fold change) of overlapping upregulated and downregulated genes associated with *Acinetobacter*. *Os04g0659300* (*OsRLK*) meets criteria for high transcript abundance and log_2_(fold change) > 2. Source data

To identify plant genes associated with *Acinetobacter*, we compared root transcriptomes of paternal wild rice grown in non-sterilized soil (WN) versus sterilized soil (W), paternal wild rice grown in sterilized soil inoculated with the *Acinetobacter* SynCom (WA) versus sterilized soil (W). Kyoto Encyclopedia of Genes and Genomes (KEGG) comparative analysis revealed significant pathways ordered by proportion of differentially expressed genes (DEGs). In WN versus W, the DEGs were enriched in betalain biosynthesis, flavonoid biosynthesis and nitrogen metabolism (Fig. 5a), while in WA vs W, enriched pathways comprised nitrogen metabolism and biosynthesis of various plant secondary metabolites (Fig. 5b). Nitrogen metabolism was the only pathway common to both comparisons. Fourteen reported NUE-related DEGs, including *OsNRT2.3a*, *NRT2.2*, *OsNAR2.1*, *OsNPF7.9*, *OsDREB1C* and *OsAMT1;3* were identified (Fig. 5c and Supplementary Table 6).

A total of 2,494 upregulated and 2,661 downregulated genes were shared between WN vs W and WA vs W comparisons (Fig. 5d). Within the *Acinetobacter*-associated 0.64 Mbp region on Chr 4, there were 16 upregulated genes and 3 downregulated genes. Among these, only the receptor-like kinase-encoding gene *OsRLK* in paternal wild rice met the criteria of high expression with log_2_(fold change) > 2 (*q*-value < 0.05) in both comparisons (Fig. 5e).

To validate these findings, *OsRLK* from wild rice was cloned to generate overexpression lines for testing interactions with the *Acinetobacter* synthetic community. Southern blot analysis confirmed one or two copies of the *OsRLK* expression cassette in six transformants (Y165, Y166, Y168, Y172, Y174 and Y175; Supplementary Fig. 1), and two single-copy lines were selected for subsequent functional analyses.

### Interaction of *Acinetobacter* SynCom with *OsRLK* rice lines

**Fig. 6:**
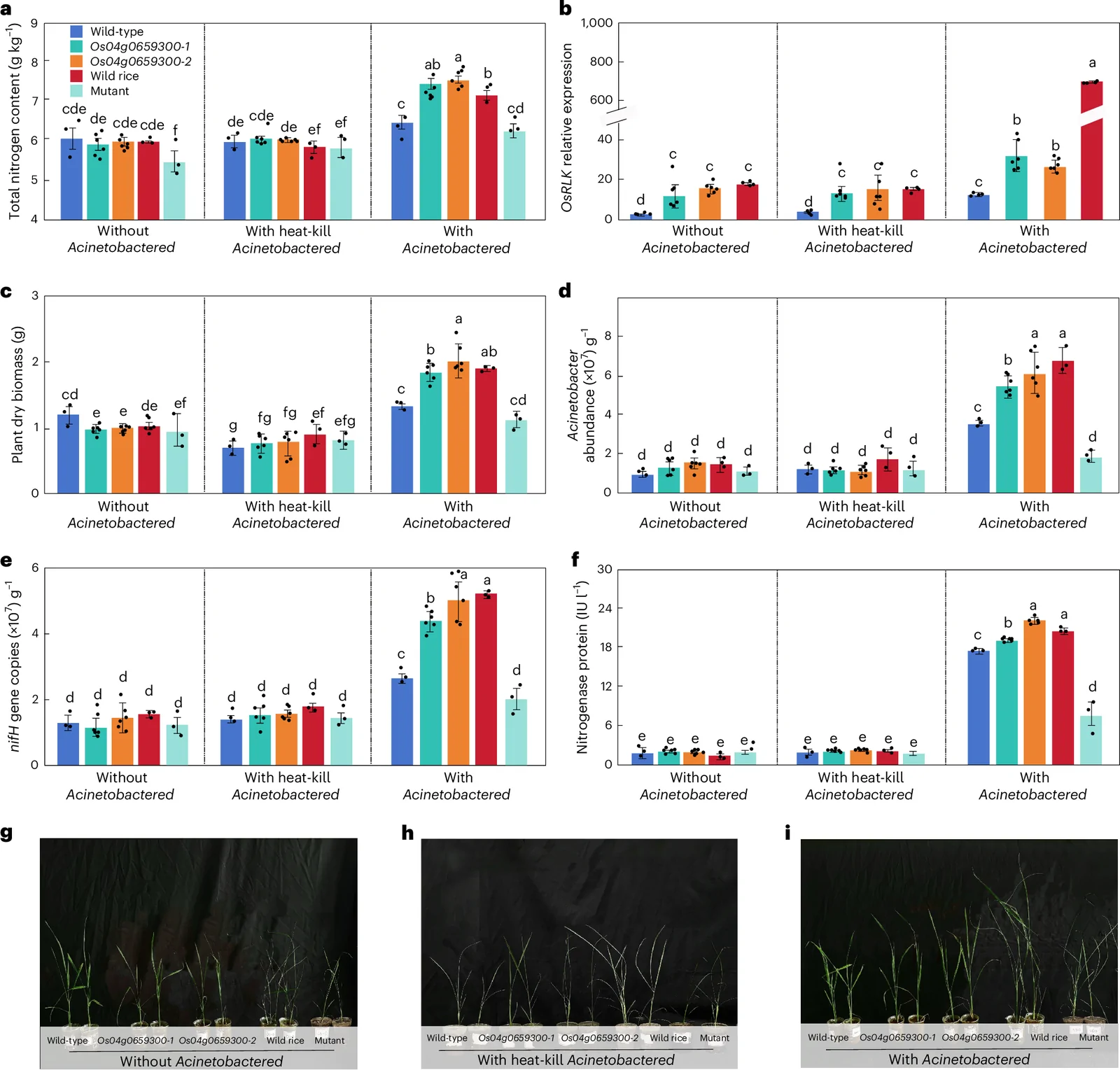
Regulatory effect of genetically modified rice on *Acinetobacter*. **a**,**c**, Total nitrogen content (**a**) and plant dry biomass (**c**) of wild-type (1504), *OsRLK*-overexpressing transgenic rice, *OsRLK*-knockout mutants (including mutant strains with three distinct mutation types, mutant-1, mutant-2 and mutant-3) and wild rice (*O. rufipogon*) with, without SynCom and with a heat-killed SynCom inoculation. Significant differences are marked by different letters (one-way analysis of variance (ANOVA), *P* < 0.05, wild-type, wild rice and mutants *n* = 3; *OsRLK*-1-overexpressing transgenic rice *n* = 6; *OsRLK*-2-overexpressing transgenic rice *n* = 6). **b**, Relative expression of *Os04g0659300* in wild-type rice, wild rice and *Os04g0659300*-overexpressing transgenic rice with, without synthetic community (SynCom) and with a heat-killed SynCom inoculation. Statistical significance of differences between genotypes is indicated by different letters (one-way ANOVA, *P* < 0.05), where wild-type and wild rice have *n* = 4; *OsRLK*-1-overexpressing transgenic rice *n* = 6; *OsRLK*-2-overexpressing transgenic rice *n* = 6. **d**–**f**, *Acinetobacter* abundance (**d**), *nifH* abundance (**e**) and nitrogenase protein (**f**) in cultivated rice 1504 (wild-type), *OsRLK*-overexpressing transgenic rice, *OsRLK*-knockout mutant and wild rice with, without SynCom and with a heat-killed SynCom inoculation. The unit of nitrogenase protein, IU l^−1^, indicates how many micromoles of nitrogen gas are converted into ammonia per minute per litre of the enzyme solution. Differences between genotypes are indicated by different letters (one-way ANOVA, *P* < 0.05; wild-type, wild rice and mutant *n* = 3; *OsRLK*-1 transgenic rice *n* = 6; *OsRLK*-2 transgenic rice *n* = 6). Data are presented as mean ± s.d. **g**–**i**, Plant growth in wild-type and *Os04g0659300*-overexpressing transgenic rice without (**g**), with SynCom (**h**) and with a heat-killed (**i**) SynCom inoculation. Source data

Wild-type *indica* rice (1504), *OsRLK*-overexpressing lines, *OsRLK*-knockout lines and wild rice, inoculated or not with *Acinetobacter* SynCom and inoculated with a heat-killed *Acinetobacter* SynCom were evaluated. Total nitrogen (TN) levels were significantly higher in SynCom-inoculated wild rice and *OsRLK*-overexpressing lines, whereas no significant differences occurred among these rice lines without and with a heat-killed SynCom (Fig. 6a, *P* < 0.05). Gene expression was significantly higher in SynCom-inoculated transgenic and wild rice compared with non-inoculated and with heat-killed SynCom plants (Fig. 6b). Furthermore, *OsRLK* expression was consistently higher in transgenic rice than in wild-type rice (1504) under all treatments. Similarly, plant dry biomass increased in SynCom-inoculated wild and *OsRLK*-overexpressing lines compared with wild-type and *OsRLK*-knockout lines, but did not differ among lines under a heat-killed SynCom treatment (Fig. 6c).

To further assess *OsRLK* effects on *Acinetobacter* SynCom function, we quantified *Acinetobacter* and *nifH* abundances by quantitative real-time PCR (qPCR), and quantified nitrogenase protein abundance by an enzyme-linked immunosorbent assay (ELISA) kit. Without SynCom, traits and growth did not differ among wild-type (1504), *OsRLK*-overexpressing lines, *OsRLK*-knockout lines and wild rice (Fig. 6d–f, *P* < 0.05). After 30 days with SynCom, *Acinetobacter*, *nifH* and nitrogenase protein abundance ranked *OsRLK-1* and wild rice > *OsRLK-2* > wild-type > *OsRLK*-knockout mutant lines (Fig. 6d–f, *P* < 0.05). Plant growth was comparable across all lines without SynCom inoculation (Fig. 6g,h) but was enhanced in *OsRLK*-overexpressing lines and wild rice grown with SynCom inoculation (Fig. 6i).

To compare agronomic traits between the wild-type and *OsRLK* overexpression lines, we conducted field trials and evaluated yield-related traits. *OsRLK* overexpression lines significantly increased effective panicle number, seed-setting rate, thousand-grain weight and grain yield per plant (Supplementary Table 7, *P* < 0.05). In contrast, no significant differences were observed in panicle length, grain length or grain width between the overexpression lines and the wild type.

**Extended Data Fig. 7:**
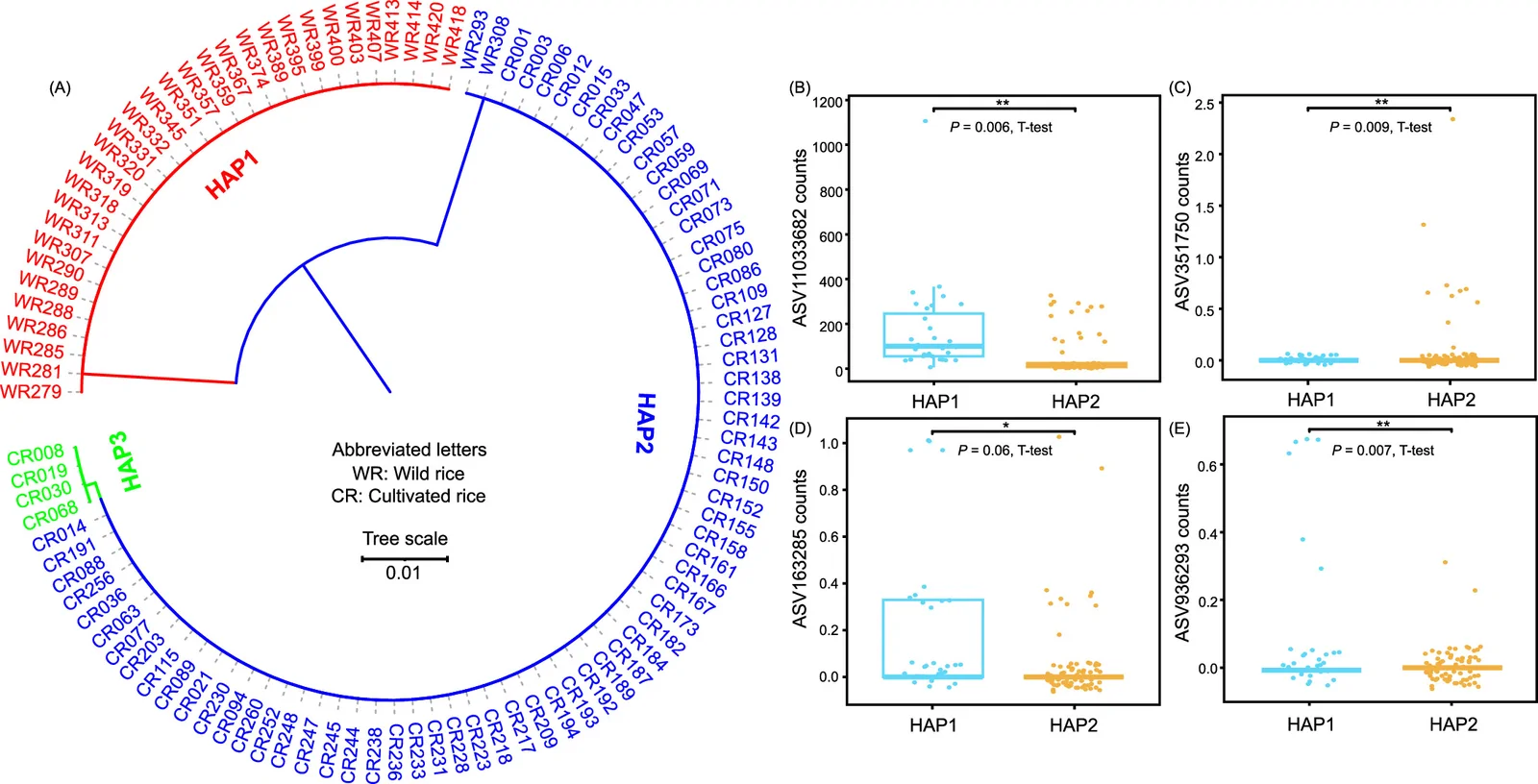
Phylogenetic tree and haplotype-phenotype analysis of *OsRLK*. (**A**) Phylogenetic tree of *OsRLK* genes in rice germplasms constructed using the Neighbor-Joining method based on full-length sequence alignments of 105 *OsRLK* genes from three haplotypes. Red, blue, and green branches represent haplotype 1 (HAP1), haplotype 2 (HAP2), and haplotype 3 (HAP3), respectively. (**B**) *Acinetobacter* (ASV1033682), (**C**) *Acinetobacter* (ASV351750), (**D**) *Acinetobacter* (ASV163285), (**E**) *Acinetobacter* (ASV936293) sequence counts among the three haplotypes. See Supplementary Table 8 for detailed data. T-test, *P* < 0.05, HAP1 functional variant have n = 31; HAP2 functional variant n = 74. Source data

To analyse natural variation of *OsRLK* and its role in *Acinetobacter* enrichment, we aligned *OsRLK* sequences from 382 rice accessions (including wild rice and cultivated rice), revealing significant differences between wild and cultivated rice accessions (Supplementary Table 4). Coding-region sequencing of 105 accessions (33 wild, 72 cultivated) identified 28 single-nucleotide polymorphisms (SNPs) and 2 indels, defining 3 haplotypes: HAP1 (31 wild), HAP2 (2 wild, 68 cultivated) and HAP3 (4 cultivated) (Supplementary Fig. 2). Amino acid alignment showed 18 variations between HAP1 and HAP2, but none between HAP2 and HAP3, indicating 2 main functional variants (Supplementary Fig. 3). Phylogenetic analysis based on *OsRLK* coding sequences from 105 rice accessions showed clear separation of HAP1 and HAP2, while HAP2 and HAP3 were closely related (Extended Data Fig. 7a). In addition, *Acinetobacter* (ASV1033682, ASV163285, ASV1936293) abundance was significantly higher in the HAP1 functional variant than in HAP2, with *OsRLK* in wild rice belonging to HAP1 and *OsRLK* in cultivated rice to HAP2 (Extended Data Fig. 7b,d,e and Supplementary Table 8).

## Discussion

This study shows that core rhizosphere bacteria from parental wild rice can be reinstated in RIL progeny under field conditions, supporting a genetic basis for microbiome assembly. This extends previous associations between plant loci and rhizosphere community composition in *Zea mays*^18^, *Setaria italica*^19^, tomato^12^, barley^11^ and *Triticum aestivum*^13^ by identifying the *M-*gene receptor-like kinase-encoding *OsRLK* and demonstrating its combinatorial beneficial effects with an *Acinetobacter* SynCom on NUE. Consistent with our previous findings that rice domestication reduced nitrogen-fixing functions and increased N_2_O-related genes in rice rhizosphere^15^, and that *indica*-associated microbiota have greater nitrogen-metabolism potential than *japonica*^20^, our findings indicate that wild-rice-based hybridization can partially restore beneficial microbial functions lost during domestication.

Beyond environmental filtering, our findings show that host genetic variation can influence the reassembly of functionally relevant microbial communities, suggesting that microbiome traits may be partially inherited and restored through breeding.

Although RILs are not cultivated accessions, they retain maternal cultivated genetic backgrounds while enabling recruitment of parental wild-rice core bacteria. They, therefore provide a genetic framework for restoring wild-derived microbial functions, including improved nitrogen acquisition and reduced greenhouse gas emissions, into modern rice breeding and potentially recovering host–microbe interactions disrupted during domestication.

Specific paternal wild loci on chromosomes 8, 11 and 12 were associated with multiple core bacterial taxa, highlighting the role of host genetics in microbiome assembly. However, loci for several core taxa could not be precisely mapped, potentially owing to QTL limitations^21^ or the absence of environmental stresses that favour their recruitment. For example, *Gaiella*, *Paenarthrobacter* and *Nocardioides* are enriched under salinity, heavy metal and cold stress^22–26^. Thus, environmental cues required for gene positioning in plants^27,28^ may also influence genetic control of rhizosphere microbiome assembly. Notably, QTLs for nitrogen metabolism-related core bacteria overlapped with reported rice NUE-associated genes, including *OsAMT1;3*(ref. ^29^), which mediates ammonium uptake and associated nitrate–ammonium dynamics that alters root metabolites^21^, and shape rhizomicrobiome recruitment.

Comparing domestication loci with NUE-associated core-taxon QTLs suggests that microbiome assembly has been shaped by both domestication and natural selection. Among candidate genes, the receptor-like kinase-encoding gene *OsRLK*^30,31^ emerged as a strong candidate based on its association with *Acinetobacter*, expression pattern and divergence between wild and cultivated rice. Recent transcriptomic analyses implicate *OsRLK* in immune regulatory networks^32^, while DUF26/CRK receptor-like kinases have been linked to symbiotic nitrogen fixation in legumes^33^. Although its molecular mechanisms remain unsolved, we hypothesize that *OsRLK* regulates root exudation, thereby modifying the rhizosphere chemical environment and recruitment of beneficial microbiota. Future studies integrating root exudate metabolomics with host transcriptomics and microbial functional analyses will help identify the molecular signals and metabolic pathways underlying *OsRLK*-mediated microbiome recruitment.

The shattering gene *Sh4*, a major domestication locus, is located close to *OsRLK* (34.2 Mb), raising the possibility that favourable *OsRLK* alleles were indirectly counterselected through linkage with *Sh4* (ref. ^34^). Their genetic distance (~2 centimorgans (cM)) suggests that this linkage could be resolved through recombination, enabling introgression of *OsRLK* through fine mapping and marker-assisted selection while minimizing linkage drag. Genome editing could provide an alternative route to deploy beneficial alleles^35^. However, practical application of these strategies requires further validation and optimization. This association may reflect retained interactions between plant loci and beneficial microbes rather than direct selection on nitrogen traits and may involve trade-offs, including immune regulation. Nevertheless, our findings provide an experimental basis for *M-*gene-based strategies to improve NUE by restoring beneficial microbiome functions, extending beyond rewilding towards microbiome-driven crop improvement.

Our study provides field-based evidence that beneficial microbiome functions lost during rice domestication can be recovered through inheritance of wild-rice genetic loci. Moving beyond correlation-based studies, we identify host loci that enable the re-domestication of beneficial microbiota and their NUE-related functions. We therefore propose ‘microbiome re-domestication’ as a genetic process whereby wild-rice alleles restore beneficial plant–microbiome interactions lost during domestication. Unlike microbiome rewilding, which primarily reintroduces wild microbial diversity, re-domestication uses host genetic variation to restore these interactions, providing a framework for microbiome-guided breeding of nutrient-efficient crops.

## Methods

The experimental framework consists of three parts (Fig. 1): First, phenomenon identification, where we mapped genetic loci linked to wild-rice core rhizosphere microbiota and their overlap with domestication loci in RILs (Fig. 1a,b). Second, mechanism elucidation, using transcriptome analyses to identify plant genes regulating microbial interactions (Fig. 1c,d). Third, functional validation, where we engineered transgenic rice and tested a synthetic *Acinetobacter* community to assess their role in nitrogen-use efficiency (Fig. 1e,f). This integrative approach combines genetic mapping, transcriptomics and functional validation to reveal gene–microbiota interactions enhancing nitrogen utilization.

### Identification of core bacterial groups in the rhizosphere of wild rice and linkage analysis

#### Plant material and growth conditions

Chinese Dongxiang common wild rice (*Oryza rufipogon*), representing the northernmost naturally distributed wild-rice germplasm, was used as paternal parent. This genotype, *O. rufipogon* Dongxiang, possesses cold resistance loci and nutrient deficiency resistance loci. Two F4 RIL populations, each consisting of 100 lines, were developed by crossing two *O. sativa* cultivars (female parents *O. sativa* L. ssp. *japonica* [Longgeng 31] and *O. sativa* L. ssp. *indica* [Huanghuazhan]) with male parent *O. rufipogon* Dongxiang.

Experimental plots for the parental lines and the two RIL populations were established at the demonstration base of the Northeast Institute of Geography and Agroecology, Chinese Academy of Sciences, located at the National Agricultural Station in Liaoheyuan (42° 54’ 15” N, 125° 26’ 10” E), Jilin Province, China. This site has temperate monsoonal climate and black soil, as classified by the United States Department of Agriculture (USDA) soil taxonomy.

Rice seeds from each RIL population and the parental lines were thoroughly mixed, and seeds were randomly selected. These seeds were soaked in tap water for 24 h, then disinfected with 70% ethanol followed by 2% sodium hypochlorite for 20 min. After disinfection, the seeds were germinated at 30 °C for 48 h. The germinated seeds were sown in 448-cell seedling trays (26 mm height, 16.5 mm diameter), with one seed per cell. One tray was used for each parental line and three trays for each RIL population.

One-month-old seedlings were transplanted to the field on 10 May 2022, with rows spaced 3–40 cm apart and one seedling per hill. The field was flooded to a depth of 10 cm until 10 days before full heading. Sampling occurred from 26–30 September 2022. To minimize border effects and variety interactions, plants were collected from the central areas of each plot. For microbiome and genome sequencing, 6 plants from the parental lines and 100 plants from each RIL population were randomly selected. Rhizosphere soil was collected for bacteria amplicon sequencing following established protocols^15^.

#### Rhizosphere microbial community

We assessed the microbial community composition in rice rhizosphere by 16S rRNA gene amplicon sequence. DNA was extracted from 0.5 g of rhizosphere soil using the Fast DNA SPIN kit (MP Biomedicals). The V3–V4 hypervariable regions of bacterial 16S rRNA gene were amplified using primers 338F (5’-ACTCCTACGGGAGGCAGCA-3’) and 806R (5’-GGACTACHVGGGTWTCTAAT-3’). The thermal cycling conditions included: initial denaturation at 98 °C for 8 min; 25 cycles of denaturation at 98 °C for 15 s, annealing at 55 °C for 30 s and extension at 72 °C for 30 s, followed by a final extension at 72 °C for 10 min. The PCR amplicons were purified using Agencourt AMPure Beads (Beckman Coulter), quantified and subjected to 250-bp paired-end sequencing on the Illumina NovaSeq platform. A total of 34,356,465 16S rRNA gene amplicon read pairs were generated from 424 samples. Raw reads were filtered using Trimmomatic v.0.33, and primer sequences were removed with cutadapt 1.9.1, resulting in 34,177,527 clean read pairs^36^. These clean reads were then denoised using the Dada2 plugin in QIIME2, producing amplicon sequence variants (ASVs)^37^. ASV taxonomic classification was performed on the basis of the Naive Bayes classifier in QIIME2 using the SILVA database (v.138.1)^38^. ASVs identified as non-bacterial, chloroplast or mitochondrial were excluded. The FAPROTAX database was utilized to predict the potential functions of the core rhizobacteriome^16^. The gjam package in R (v.4.1.2) facilitated the identification of the core rhizobacteriome, consisting of microbes with regression coefficients that were positive and significantly different from zero across the populations. The model was run with a total of 9,000 iterations, of which the first 3,000 were discarded as burn-in to ensure convergence.

#### Plant physiological properties and nitrogen content

Leaves were used for chlorophyll concentration analysis, and the remaining plant material was used to measure fresh and dry weights of shoots and roots, as well as nitrogen content. Replicate counts varied by analysis: *n* = 6 for parental lines and bulk soil, *n* = 3 per RIL population sample. Rice parameters including dry weight, plant height, root length, main spike length, chlorophyll content and nitrogen content were determined. Plant height, main spike length and main root length were measured using standard rulers, and ground diameter was assessed using Vernier calipers. Fresh shoots and roots were weighed and then dried at 60 °C for 48 h to determine their dry weights. The dried shoots and roots were ground together and analysed as a composite sample to measure plant nitrogen content using a continuous-flow analyser (AutoAnalyzer3 Continuous-Flow Analyzer, Bran Luebbe). Chlorophyll was extracted from 0.1 g of fresh leaves using 95% ethanol, with three replicates per accession. Chlorophyll concentration (Chl) in the leaves was quantified using a spectrophotometer, adhering to a previously described method^39^. The equations defining the relationship between chlorophyll concentration and optical density are as follows:

1 $${{D}}665=83.31{Ca}+18.60{Cb}$$

2 $${{D}}649=24.54{Ca}+44.24{Cb}$$

3 $$C{\rm{T}}=Ca+Cb$$

where *D*_665_ and *D*_649_ represent the optical densities at wavelengths of 665 nm and 649 nm, respectively; *Ca*, *Cb* and *C*T denote the concentrations of chlorophyll *a*, chlorophyll *b* and total chlorophyll, respectively. The coefficients 83.31 and 18.60 are the specific absorptions for chlorophyll *a* and chlorophyll *b* at 665 nm, respectively; 24.54 and 44.24 are the specific absorptions at 649 nm.

### Plant DNA extraction and SNP calling for two RIL populations

Plant genomic DNA was extracted from fresh leaves of the two rice populations at rice maturity stage. Genomic DNA was extracted from young leaves using a cetyltrimethylam-monium bromide (CTAB)-based protocol^40^. The concentration and quality of the total genomic DNA were assessed using a NanoDrop2000 spectrophotometer (Thermo Fisher). DNA libraries (350 bp) for Illumina/BGI sequencing were constructed for each accession according to manufacturer specifications. After DNA library construction, sequencing was performed on an Illumina HiSeq XTen/NovaSeq/BGI platform by a commercial service (Biomarker Technologies), with 150-bp read lengths. The raw reads were further processed with the bioinformatic pipeline tool BMKCloud (www.biocloud.net) online platform. Raw data (raw reads) of fastq were first processed through fastp software. In this step, clean data (clean reads) were obtained by removing reads containing adapter, reads containing ploy-N and low-quality reads (quality score <20) from raw data. At the same time, Q20, Q30, GC content and sequence duplication level of the clean data were calculated. All downstream analyses were based on clean data with high quality. Raw sequences were transformed into clean reads after data processing. Clean reads were then mapped to the *Oryza sativa* Nipponbare MSU 7.0 genome using Burrows–Wheeler Aligner software^41^, as this genome is widely recognized for its high-quality assembly and annotation, providing a reliable reference essential for accurate mapping and meaningful genetic insights. Duplicate reads were removed using Samtools^42^, followed by local realignment and base recalibration with Genome Analysis Toolkit (GATK)^43^. Genotypes for 76 *O. sativa* L. ssp. *japonica* and 90 *O. sativa* L. ssp. *indica* accessions were determined using a combined GATK and Samtools SNP calling analysis under default settings.

SNPs exhibiting polymorphism between the two parentals were classified into eight segregation patterns, with only SNPs following the aa × bb pattern selected for further analysis. Recombination breakpoints were identified using a sliding-window approach to construct a physical bin map, with bins serving as markers for developing an ultra-high-density genetic map. These markers were grouped into linkage groups based on their physical locations on the *Oryza sativa* Nipponbare whole genome. To ensure linkage group integrity, modified logarithm of odds (MLOD) scores between markers were calculated. Markers with MLOD scores below 5 were excluded from ordering. The HighMap strategy^44^ was used to order SNP markers and correct genotyping errors within linkage groups. Recombinant frequencies and LOD scores were determined through two-point analysis to infer linkage phases. Marker ordering was performed iteratively using simulated annealing algorithms^45,46^. The SMOOTH strategy^47^ was applied to correct errors based on parental genotype contributions, and a *k*-nearest neighbour algorithm was used to impute missing genotypes^48^. Skewed markers were integrated into the map using a multipoint maximum likelihood approach, and map distances were estimated using the Kosambi mapping function^49^.

#### QTL analysis

After analysing the core rhizosphere bacterial community of paternal wild-rice accessions and excluding the core microbiomes of bulk soil and cultivated parental lines, ASVs exclusively selected by the paternal wild-rice microbiome were mapped within the RIL populations. The QTL analysis linking selected core ASV features with plant loci was conducted using the R package R/qtl2 (ref. ^50^), with genotypic and phenotypic data obtained from the same individual plants. Interval mapping using the expectation-maximization (EM) algorithm and a single-QTL model was implemented. A randomized permutation test with 1,000 permutations was performed to assess the distribution of LOD scores. The threshold for identifying significant LOD peaks was set at the 95th percentile using a Bayesian credible interval with a probability of *P* = 0.95.

### Analysis of overlap between core bacterial taxa-related QTLs and domestication loci

#### Screening of rice domestication loci

To explore rice domestication loci, 117 wild rice (*O. rufipogon*) and 265 cultivated rice (*O. sativa* L. ssp. *japonica* and *O. sativa* L. ssp. *indica*) accessions were planted in the same field on 15 May 2023. The field was maintained with a water depth of 10 cm until 10 days before the full heading. Whole plants, including roots, were harvested between 20 and 30 September 2023. To minimize border effects and interference between accessions, plants of the same variety were collected from the centre of each planting area. Genomic sequences for 260 rice accessions were obtained from a published study^51^ (https://www.ncbi.nlm.nih.gov/sra/?term=PRJEB6180), while the remaining 122 accessions were sequenced in this study (Supplementary Table 4).

Plant genomic DNA was extracted from fresh leaves, and genomic sequences were processed as described in Experiment II. SNPs were identified within the 382 accessions using the HaplotypeCaller module in GATK (v.3.8)^43^. These SNPs were filtered using the following parameters: QD < 2.0||MQ < 40.0||FS > 60.0||QUAL < 30.0||MQrankSum < −12.5||ReadPosRankSum < −8.0, -clusterSize 2, -clusterWindowSize 5. Only SNPs with a minor allele frequency greater than 5% and less than 80% missing data were retained. A total of 1,489,886 high-quality SNPs were obtained. Nucleotide diversity (*π*) was calculated for each SNP using vcftools^52^, employing a 100-kb sliding window with a 10-kb step size for each subpopulation. The genetic diversity ratio (*π*_wild_/*π*_cultivar_) between wild and cultivated rice was quantified across the genome, and a threshold value was determined using permutation tests. This ratio was used to identify domestication loci in the *indica* and *japonica* varieties population. Comparative analysis of the 20 core ASVs common to wild, RIL-J and RIL-I population-associated QTLs and plant domestication loci was then performed. Overlapping confidence intervals between core ASV-based QTLs and domesticated loci, where the genetic diversity ratio exceeded the threshold, indicate domestication selection to those loci.

### Transcriptome analysis of the impact of wild-rice rhizosphere microbiome on plants

#### Pot experiment

To assess whether the rhizosphere microbiome of paternal wild-rice accessions enhances plant growth and development, Chinese Dongxiang common wild-rice accessions were planted in sterilized soil (W) and non-sterilized soil (WN). Rice seeds were surface sterilized with 2.5% NaClO and germinated in deionized water for 5 days at 26 °C in darkness. Seedlings were then transferred to sterilized and non-sterilized black soil, collected from the Northeast Institute of Geography and Agroecology experiment station and sterilized with gamma irradiation at 25 kGy. Rice plants were grown under controlled environmental conditions: 15 h of light at 35 °C and 9 h of darkness at 30 °C, with 70–80% relative humidity.

#### Transcriptome analyses of wild rice

Root samples were collected at 30 days post transplantation following previously described methods. Samples were washed with demi water and immediately frozen in liquid nitrogen for RNA extraction using a Promega RNA extraction kit (Promega, LS1040). The quality and concentration of the extracted RNA were assessed using an RNA Nano 6000 Assay kit on a Bioanalyzer 2100 system (Agilent). Total RNA was then used for complementary (c)DNA library preparation, involving mRNA isolation, fragmentation and reverse transcription. Double-stranded cDNA was synthesized, size-selected and amplified via PCR. The quality of the library was verified using the Agilent Bioanalyzer 2100 system. Index-coded samples were clustered and sequenced on an Illumina HiSeq XTen platform (Illumina) on a cBot Cluster Generation system following manufacturer instructions. Sequencing was performed on an Illumina Novaseq platform, producing 150-bp paired-end reads.

Raw fastq data were processed to remove adapters, poly-N sequences and low-quality reads using Perl scripts. Clean reads were aligned to the reference genome using Hisat2 v.2.0.5. The assembly of mapped reads for each sample was performed with StringTie (v.1.3.3b) using a network flow algorithm, which quantified full-length transcripts and splice variants for each gene locus^53^. Differential expression analysis between the two soil treatments was conducted using edgeR. Genes exhibiting differential expression between sterilized and non-sterilized soil-planted wild rice were identified using corrected *P* values (*q*-values) < 0.05, controlled using the Benjamini–Hochberg method, with a significance threshold set at a *q*-value of 0.05 and an absolute fold change of 2.0. Gene Ontology (GO) enrichment analysis on differentially expressed genes was performed using the clusterProfiler R package, considering GO terms with *q*-values < 0.05 as significantly enriched. KEGG pathway annotations were facilitated using KAAS (KEGG Automatic Annotation Server: http://www.genome.jp/kegg/kaas/), and statistical enrichment of differentially expressed genes in KEGG pathways was tested using the same package.

#### Isolation of core bacterial taxa and construction of synthetic community

Following our findings that strong selective sweeps in domestication loci are associated with nitrogen fixation-related core microbes *Acinetobacter* ASV6549, this bacterium was notably linked to rice candidate gene segments related to nitrogen utilization efficiency. Given its high additive effect difference (ADD) value and phenotypic variance explained (PVE) value, *Acinetobacter* was selected for further validation as a core nitrogen-fixing bacterium in the rhizosphere of paternal wild-rice accessions, potentially influencing plant loci associated with nitrogen use efficiency.

*Acinetobacter* was isolated from rhizosphere soil samples collected from paternal wild rice. Approximately 0.5 g of rhizosphere soil samples were homogenized in distilled water, and the resultant solution was diluted and cultured on medium at 28 °C for 3 days. The medium includes potassium nitrate (1.0 g l^−1^), asparagine (1.0 g ^−1^), bromothymol blue (BTB) (0.05 g l^−1^), sodium citrate (8.5 g l^−1^), magnesium sulfate heptahydrate (1.0 g l^−1^), ferric chloride hexahydrate (0.05 g l^−1^), potassium dihydrogen phosphate (1.0 g l^−1^) and calcium chloride dihydrate (0.2 g l^−1^). After four rounds of re-plating on culture medium plates, individual colonies were selected and identified through 16S rRNA amplicon sequencing, following the methods described above. qPCR of the *nifH* gene was conducted to verify the nitrogen fixation capability of the isolated strains, employing primers nifHF (5’-AAAGGYGGWATCGGYAARTCCACCAC-3’) and nifHR (5’-TTGTTSGCSGCRTACATSGCCATCAT-3’). Plasmid DNA extracted from microbiomes harbouring *nifH* gene from rhizosphere samples of paternal wild-rice accessions was used to generate standard curves, which were then cloned into vectors. The standard curve involved 10-fold serial dilutions ranging from 10^1^ to 10^7^. High reaction efficiencies (90–105%) were achieved for gene quantification, with *R*^2^ values exceeding 0.99. qPCR analyses were performed in biological triplicates, each with 3 technical replicates. Concurrently, a minus-template qPCR using distilled water instead of rhizosphere DNA was conducted as a control. On the basis of their nitrogen fixation capabilities, 9 strains of *Acinetobacter* sp., including two *A. baumannii* strains, *A. seifertii* strain, *A. halotolerans* strain, *A. sichuanensis* strain, *A. schindleri* strain, *A. geminorum* strain, *A. defluvii* strain and *A. lactucae* strain were isolated from the rhizosphere soil of wild rice (Extended Data Fig. 5). PCR amplification was used to confirm the isolated strains. These strains were cultured overnight in tryptic soy broth medium at 37 °C. Cells from each strain were then washed and combined in equal concentrations to construct a synthetic community of *Acinetobacter*.

### Transcriptomic analysis of the impact of *Acinetobacter* on plants

To identify candidate genes regulating the genus *Acinetobacter*, we planted Chinese Dongxiang common wild rice in two soil conditions: sterilized soil inoculated with *Acinetobacter* synthetic bacterial communities (WA) and sterilized soil (W) only. *Acinetobacter* synthetic bacterial communities (1.0 ml, with an optical density at 600 nm (OD_600_) of 0.5) were added to 100 ml of sterilized water and combined with 100 g of black soil (resulting in a final concentration of 10^6^ colony-forming units (c.f.u.s) g^−1^) for planting wild rice. The experimental design, including rice materials, seed sterilization and planting management, followed the protocol described above for the pot experiment assessing transcriptome analyses of wild rice. The transcriptomic analyses adhered to the methods described above.

### Construction of genetically modified rice material

**Extended Data Fig. 8:**
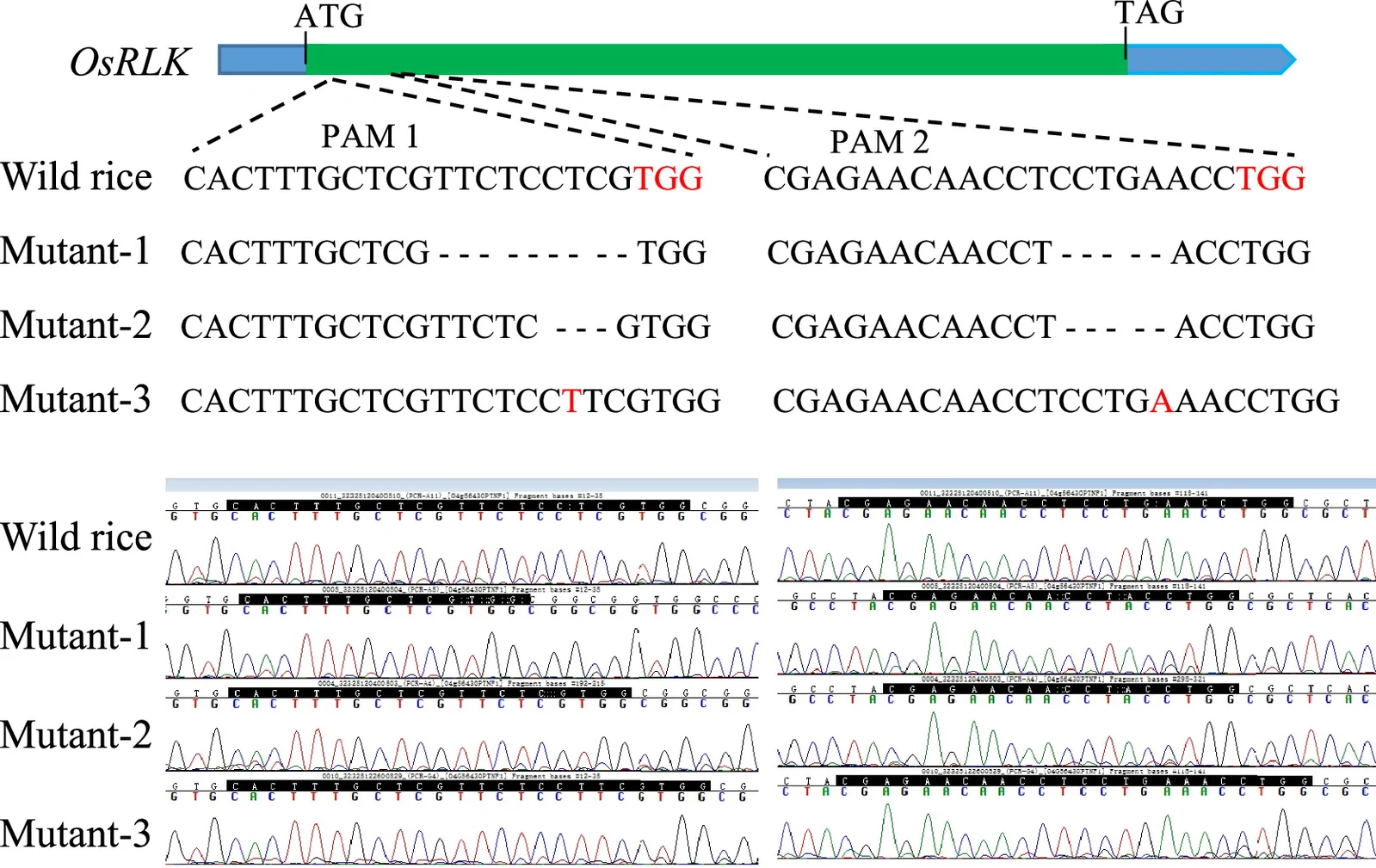
Identification of *OsRLK* mutants. (Top) *OsRLK* mutation sites and their mutation types. (Bottom) Sequencing chromatograms of wild rice *OsRLK* and its mutants.

The selection of candidate genes was guided by three criteria: First, candidate genes must exhibit simultaneous upregulation in comparisons of both WN with W, and WA with W; second, they should demonstrate a log_2_(fold change) > 2; third, the transcript abundance of candidate genes should exceed the average transcript abundance of all genes under the same conditions. On the basis of these criteria, *Os04g0659300* (*OsRLK*) was selected as a candidate gene on chromosome 4 for regulating ASV6549 (*Acinetobacter*). To test the hypothesis that *OsRLK* contributes to nitrogen utilization efficiency and influences *Acinetobacter*, *OsRLK* overexpression lines and knockout mutants were generated using *indica* rice 1504 (wild-type) and Chinese Dongxiang wild rice (wild rice) as recipient materials, respectively. The pU1301 vector^54^ containing a maize ubiquitin promoter was used for the overexpression studies. To construct the overexpression vector of *OsRLK*, the genomic sequence of *OsRLK* from Chinese Dongxiang wild rice was amplified and cloned into pU1301. The *OsRLK* mutants were generated using CRISPR/Cas9 technology as previously described^55^. Two specific protospacer adjacent motif (PAM) sequences (PAM1: CACTTTGCTCGTTCTCCTCGTGG, PAM2: CGAGAACAACCTCCTGAACCTGG) of *OsRLK* were selected. Subsequently, the corresponding single-guide RNAs (sgRNAs) (sgRNA1: CACTTTGCTCGTTCTCCTCG, sgRNA2: CGAGAACAACCTCCTGAACC) were introduced into the pBWA (V) H2S-cas9pl (osu3) i vector backbone to construct the Cas9-*OsRLK* recombinant vector. The constructs were confirmed by sequencing, introduced into *Agrobacterium tumefaciens* strain EHA105 by electroporation, and transformed into the corresponding recipient materials by *Agrobacterium*-mediated transformation. The primers used for vector construction are listed in Supplementary Table 9. The PAM sequences of *OsRLK* mutant plants were sequenced to detect their mutation types, and homozygous mutants were selected for subsequent experiments (Extended Data Fig. 8).

For Southern blot hybridization to determine the copy number of the *OsRLK*^DY^ transgene, 10 µg of genomic DNA from both overexpressed and wild-type rice materials from 30-day-old seedlings was digested with 15 U of HindIII (NEB) for 16 h at 37 °C. The digested DNA was resolved on a 0.8% agarose gel at 30 V for 16 h, followed by capillary transfer onto a Hybond N+ nylon membrane (GE Healthcare) overnight. The blot was hybridized at 48 °C with a DNA probe complementary to the HPT gene, which encodes hygromycin B phosphotransferase (HPT) and serves as a selectable marker in genetic transformation, and then exposed to X-ray film at −80 °C for 4 h for imaging.

All transgenic plant materials were planted in Jiangxi Academy of Agricultural Sciences Biological Breeding Base at Yichun (28° 14’ 57” N, 115° 07’ 05” E) in 2024 for phenotype investigation. Each line was grown in a two-row plot with 10 plants per row, with a spacing of 16.7 cm between plants and 21.6 cm between rows. At the mature stage, effective panicle number per plant and panicle length were evaluated following the methods described previously^56^. Spikelet number per panicle, seed-setting ratio, grain length, grain width, thousand-grain weight and grain weight per plant were measured using a yield traits scorer in a high-throughput phenotyping facility^57^.

### Validation of genetically modified rice materials in regulating *Acinetobacter*

#### Experiment design

The experiment utilized overexpressed rice genotype and synthetic microbial communities to confirm the regulatory relationship between gene *OsRLK* and the *Acinetobacter* synthetic community. This synthetic microbial community and a heat-killed synthetic microbial community (used as control) were inoculated into sterilized soil alongside both overexpressed and wild-type-rice material. Each pot received 1.0 ml of synthetic communities (with an OD_600_ of 0.5) mixed with 100 g of black soil, resulting in a final concentration of 10^6^ c.f.u.s g^−1^. Rice plants were grown under controlled conditions: 35 °C with 15 h of light per day and 30 °C during 9 h of darkness, maintaining 70–80% relative humidity. Rhizosphere soil samples were collected at 30 days post inoculation (dpi) to measure *Acinetobacter* abundance, *nifH* gene abundance and nitrogenase activity. Rhizosphere soil (0.5 g) was suspended in PBS at a 1:10 (w/v) ratio, vigorously vortexed for 40 s and then centrifuged at speed 4,000 revolutions per min for 15 min. The resulting supernatant was subjected to nitrogenase protein quantification using the Soil Nitrogenase (NITS) ELISA kit (ADANTI). At 30 dpi, rice plants were harvested for further analysis, with each treatment including 5 biological replicates.

#### Samples determination

DNA was extracted from 0.5 g of rhizosphere soil samples collected at 0, 5, 15 and 30 dpi using the Fast DNA SPIN kit (MP Biomedicals). qPCR of the *Acinetobacter* gene was conducted to verify *Acinetobacter* colonization, employing primers Ac436f (5’TTT AAG CGA GGA GGA GG 3’) and Ac676r (5’ATT CTA CCA TCC TCTCCC3’)^58^. *nifH* primers and qPCR analysis was performed following the procedures outlined in Experiment Ⅴ. Leaf samples were also collected at 30 dpi to assess the expression of the *OsRLK* gene, using the RT–qPCR methods previously described^59^. In addition, dry shoots and roots were combined and ground together to form a single sample for measuring plant nitrogen content. This analysis was conducted using a continuous-flow analyser (AutoAnalyzer3 Continuous-Flow Analyzer, Bran Luebbe).

#### Haplotype analysis and phylogenetic tree construction

The natural variations in the coding region of *OsRLK* in the world-wide rice core collection of 105 accessions (including 33 accessions of wild rice and 72 accessions of cultivated rice) were obtained from sequencing. Haplotype analysis and phylogenetic tree construction were performed on the basis of the sequencing data of 105 germplasm accessions. The EMBL software CLUSTAL 2.0.12 multiple sequence alignment tool was used to perform the coding of DNA sequences and protein alignment. Phylogenetic trees were constructed using the molecular evolutionary genetics analysis version 7.0 (MEGA7) software^60^.

#### Statistical analysis

Differences in the rhizosphere bacterial community structure between the parental lines and the two RIL populations were assessed using non-metric multidimensional scaling (NMDS) based on the Bray–Curtis dissimilarity matrix in the vegan package in R (v.4.1.2). PERMANOVA was employed to evaluate the statistical significance of clustering patterns observed in the ordination plots. GJAM was conducted to determine the core bacteriome profiles and to identify core bacteriomes of different accessions recruited under similar soil conditions^61,62^. The gjam package in R (v.4.1.2) facilitated the identification of the core rhizobacteriome, consisting of microbes with regression coefficients that were positive and significantly different from zero across the populations. Visualizations, including Sankey diagrams, were created using Origin 2023. On the basis of the taxonomic classification, a species tree was inferred using GraPhlAn, which displays taxonomic data generated by other metagenomic analysis tools without branch lengths^62,63^. Correlations among plant physiological and biochemical properties were determined using Pearson’s correlation coefficients via the linKEt package in R (v.4.1.2). Mantel correlations between core microbial composition and plant physiological and biochemical data, with 9,999 permutations, were analysed using the vegan package in R (v.4.1.2)^64^.

### Reporting summary

Further information on research design is available in the Nature Portfolio Reporting Summary linked to this article.

## Supplementary information

Supplementary information Supplementary Figs. 1–3 and the uncropped gel.

Reporting Summary

Supplementary Tables 1–9 Supplementary Tables 1–9. Each supplementary table is provided as a separate worksheet, and the corresponding table title and legend are included within each worksheet.

## Source data

Source Data Fig. 2 Statistical source data, including the regulation ratios of the mapped core bacterial ASVs in the RIL-J and RIL-I populations.

Source Data Fig. 3 Statistical source data, including the QTL mapping results and network data used to construct the ASV–QTL hierarchical network.

Source Data Fig. 4 Statistical source data, including genome-wide selective sweep analyses (*π*_wild_/*π*_cultivar_ and FST) and QTL mapping data for NUE-associated core bacterial ASVs.

Source Data Fig. 5 Statistical source data, including KEGG pathway enrichment results, differential gene expression analyses and expression data for candidate genes associated with *Acinetobacter*.

Source Data Fig. 6 Statistical source data, including measurements of plant growth, total nitrogen content, *OsRLK* expression, *Acinetobacter* abundance, *nifH* abundance, nitrogenase protein and associated statistical analyses.

Source Data Extended Data Fig. 1 Statistical source data, including Bray–Curtis-based NMDS coordinates, alpha diversity indices and associated statistical analyses.

Source Data Extended Data Fig. 2 Statistical source data, including the classification of paternal wild rice-specific core ASVs and the numbers of shared and unique ASVs in the RIL-J and RIL-I populations.

Source Data Extended Data Fig. 3 Statistical source data, including plant physiological and biochemical measurements, Pearson correlation coefficients and Mantel test results.

Source Data Extended Data Fig. 4 Statistical source data, including QTL mapping results, marker positions and the genomic locations of NUE-associated candidate genes.

Source Data Extended Data Fig. 5 Statistical source data, including genotype variation data for the *OsRLK* locus across 382 rice accessions.

Source Data Extended Data Fig. 6 Statistical source data, including qPCR quantification of the *nifH* gene, nitrogenase protein measurements and 16S rRNA sequence information used for phylogenetic analysis.

Source Data Extended Data Fig. 7 Statistical source data, including *OsRLK* haplotype information, phylogenetic analysis, *Acinetobacter* sequence counts and associated statistical analyses.

## Data Availability

All data needed to evaluate the conclusions in this paper are presented in the main text and/or Supplementary Information. The 16S rRNA gene sequences were deposited in the National Center for Biotechnology Information (NCBI; https://www.ncbi.nlm.nih.gov/) under accession number PRJNA1005712. The whole-genome resequencing data of the recombinant inbred line (RIL) populations used for QTL mapping have been deposited in the NCBI SRA under accession PRJNA1507297. The whole-genome resequencing data of rice accessions 261–382 have been deposited in the NCBI SRA under accession PRJNA1347954, whereas genome resequencing data of accessions 1–260 (Excel File 4) were obtained from the published dataset PRJEB6180. The RNA-seq data have been deposited in the NCBI SRA under accession PRJNA1508715. The 16S rRNA gene sequences of the nine *Acinetobacter* strains used in this study have been deposited in GenBank under accession numbers PZ835331–PZ835339. The bacterial isolates and rice lines used in this study are available from the corresponding authors upon reasonable request and subject to standard material transfer agreements. Source data are provided with this paper.
