# Supplementary material for: Wild rice locus enables microbiome re-domestication for enhanced nitrogen-use efficiency
Source: Nat Microbiol. 2026 Sep 30;11(11):3010–24. doi: 10.1038/s41564-026-02498-x (PMC13739750; doi:10.1038/s41564-026-02498-x)
Supplement: Supplementary file 1 [file 41564_2026_2498_MOESM1_ESM.pdf]

nature microbiology

Supplementary information https://doi.org/10.1038/s41564-026-02498-x

Wild rice locus enables microbiome re-domestication for enhanced nitrogen-use efficiency

In the format provided by the authors and unedited

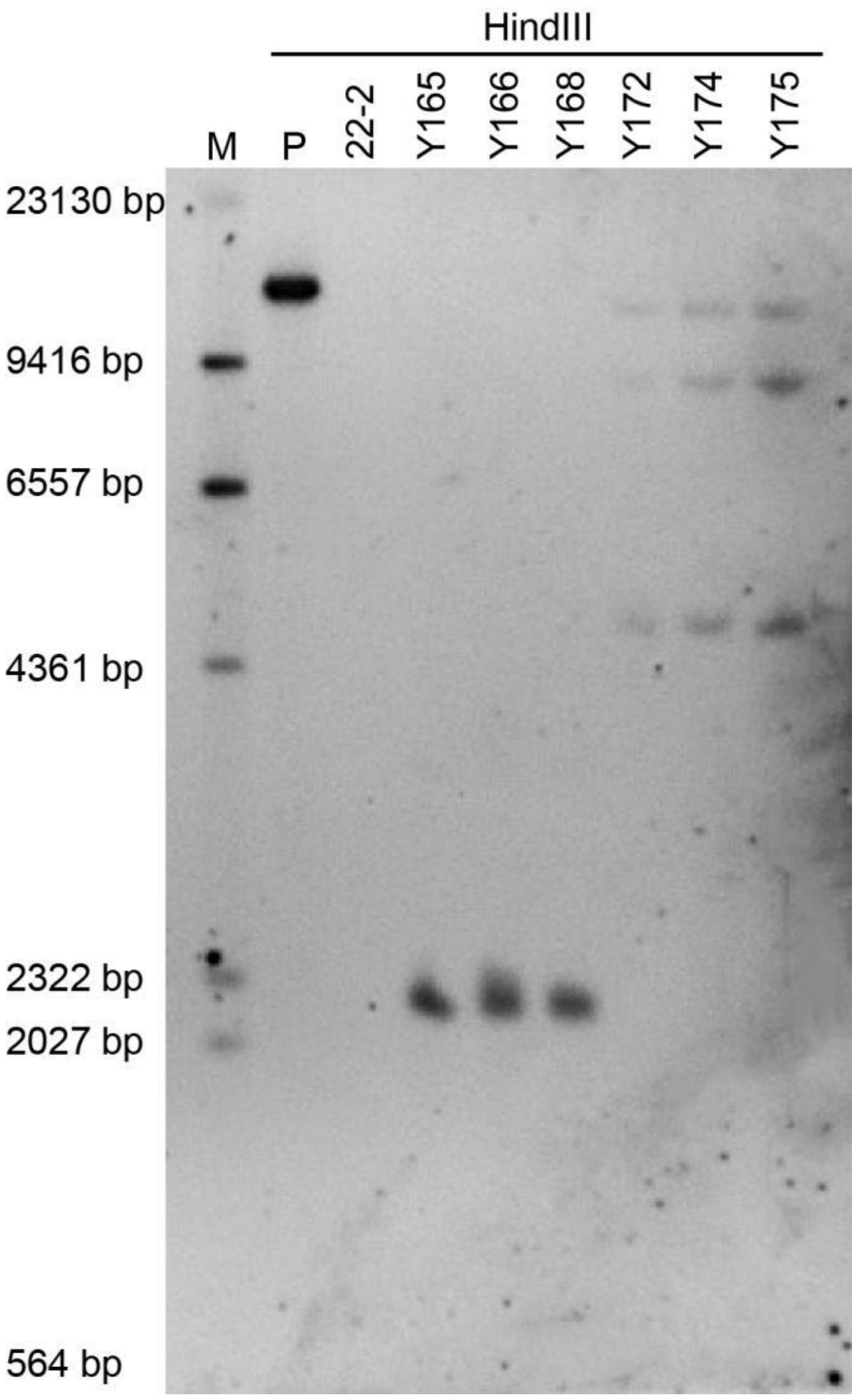

Fig. S1. Southern blot detection of the hpt gene using a 557-bp probe. HindIII: a restriction enzyme; M: DNA marker; P: PU1301 positive plasmid; 22-2: wild-type rice; Y165, Y166, Y168: *OsRLK*-1-overexpressing transgenic rice; Y172, Y174, Y175: *OsRLK*-2-overexpressing transgenic rice.

```
HAP1    ATGGCGCGGTGCACTTTGCTCGTTCTCCTCGT---GGCGGCGGTGGCccTCGTCCCGCTC
HAP2    ATGGCGCGGTGCACTTTGCTCGTTCTCCTCGTGGCGGCGGCGGTGGCggTCGTCCCGCTC
HAP3    ATGGCGCGGTGCACTTTGCTCGTTCTCCTCGTGGCGGCGGCGGTGGCggTCGTCCCGCTC

HAP1    GCCGCCGGCCAGCCGTGGGtgACCTGCGGCGACGGCACGTACaAGaAGGGGAGCGCCTAC
HAP2    GCCGCCGGCCAGCCGTGGGCTACCTGCGGCGACGGCACGTACGAGCAGGGGAGCGCCTAC
HAP3    GCCGCCGGCCAGCCGTGGGCTACCTGCGGCGACGGCACGTACGAGCAGGGGAGCGCCTAC

HAP1    GAGAACAACCTCCTGAACCTGGCgCTCACCCTCCGCGACGGCGCCTCCTCCCAGGAGATC
HAP2    GAGAACAACCTCCTGAACCTGGCCCTCACCCTCCGCGACGGCGCCTCCTCCCAGGAGATC
HAP3    GAGAACAACCTCCTGAACCTGGCCCTCACCCTCCGCGACGGCGCCTCCTCCCAGGAGATC

HAP1    CTCTTCTCCACGGGCTCCAACGGCGCCGCCCCGAACACCGTCTACGGCCTCCTCCTCTGC
HAP2    CTCTTCTCCACGGGCTCCAACGGCGCCGCCCCGAACACCGTCTACGGCCTCCTCCTCTGC
HAP3    CTCTTCTCCACGGGCTCCAACGGCGCCGCCCCGAACACCGTCTACGGCCTCCTCCTCTGC

HAP1    CGCGGCGACATCTCCCGCtCCGCCTGCTACGACTGCGGCACCAGCGTGTGGAGGGACGCC
HAP2    CGCGGCGACATCTCCCGCGCCGCCTGCTACGACTGCGGCACCAGCGTGTGGAGGGACGCC
HAP3    CGCGGCGACATCTCCCGCGCCGCCTGCTACGACTGCGGCACCAGCGTGTGGAGGGACGCC

HAP1    GGGcGCGCGTGCCaCCGCGCCAAGGACGTCGCCCTCGTCTACAACGAGTGCTACGCCCGC
HAP2    GGGAGCGCGTGCCGCCGCGCCAAGGACGTCGCCCTCGTCTACAACGAGTGCTACGCCCGC
HAP3    GGGAGCGCGTGCCGCCGCGCCAAGGACGTCGCCCTCGTCTACAACGAGTGCTACGCCCGC

HAP1    CTCTCCGACAAGGACGACTTCCTCGCCGACAAGGTcGGGCCcGGGCAGCTGACcAaCCTC
HAP2    CTCTCCGACAAGGACGACTTCCTCGCCGACAAGGTgGGGCCgGGGCAGCTGACgACCCTC
HAP3    CTCTCCGACAAGGACGACTTCCTCGCCGACAAGGTgGGGCCgGGGCAGCTGACgACCCTC

HAP1    ATGAGCAcCgCCAACATCAGCA---cCaCCGACaTCGCCGgCTACGACCGCGCGGTGACG
HAP2    ATGAGCAgCACCAACATCAGCAGCGgCGCCGACGTCGCCGCCTACGACCGCGCGGTGACG
HAP3    ATGAGCAgCACCAACATCAGCAGCGgCGCCGACGTCGCCGCCTACGACCGtGCGGTGACG

HAP1    CaGCTGCTGGCGGCCACCGCGGAGTACGCcGCGGGGGACATCGCGAGGAAGCTGTTCGCG
HAP2    CGGCTGCTGGCGGCCACCGCGGAGTACGCgGCGGGGGACATCGCGAGGAAGCTGTTCGCG
HAP3    CGGCTGCTGGCGGCCACCGCGGAGTACGCgGCGGGGGACATCGCGAGGAAGCTGTTCGCG

HAP1    ACGGGGCAGCGGGTGGGCaCCGACCCGGGGTTCCCCAACCTGTACGCGACGGCGCAGTGC
HAP2    ACGGGGCAGCGGGTGGGCGCCGACCCGGGGTTCCCCAACCTGTACGCGACGGCGCAGTGC
HAP3    ACGGGGCAGCGGGTGGGCGCCGACCCGGGGTTCCCCAACCTGTACGCGACGGCGCAGTGC

HAP1    GCGTTCGACATCACGCTGGAGGCGTGCCGCGGCTGCCTCGAGGGCCTCGTCGCCAGGTGG
HAP2    GCGTTCGACATCACGCTGGAGGCGTGCCGCGGCTGCCTCGAGGGCCTCGTCGCCAGGTGG
HAP3    GCGTTCGACATCACGCTGGAGGCGTGCCGCGGCTGCCTCGAGGGCCTCGTCGCCAGGTGG

HAP1    TGGGACACGTTCCCGGCGAACGTCGcCGGCGCCAGGATgGCCGGGCCGAGGTGCCTCCTg
HAP2    TGGGACACGTTCCCGGCGAACGTCGACGGCGCCAGGATCGCCGGGCCGAGGTGCCTCCTC
HAP3    TGGGACACGTTCCCGGCGAACGTCGACGGCGCCAGGATCGCCGGGCCGAGGTGCCTCCTC

HAP1    AGGTCGGAGGTGTAtCCGTTCTACACCGGCGCCCCGATGGTGGTGCTGCGgGAGTAG
HAP2    AGGTCGGAGGTGTACCCGTTCTACACCGGCGCCCCGATGGTGGTGCTGCGTGAGTAG
HAP3    AGGTCGGAGGTGTACCCGTTCTACACCGGCGCCCCGATGGTGGTGCTGCGTGAGTAG
```

Fig. S2. Coding DNA Sequences (CDS) of *OsRLK* from three haplotypes (HAP1, HAP2, and HAP3). CLUSTAL 2.0.12 (EMBL) was used to align the CDS. The same color indicates completely conserved nucleotide positions.

```
HAP1        MARCTLLVLLV-AAVAlVPLAAGQPWvTCGDGTYkkGSAYENNLLNLALTLRDGASSQEI
HAP2        MARCTLLVLLVAAAVAVVPLAAGQPWATCGDGTYEQGSAYENNLLNLALTLRDGASSQEI
HAP3        MARCTLLVLLVAAAVAVVPLAAGQPWATCGDGTYEQGSAYENNLLNLALTLRDGASSQEI

HAP1        LFSTGSNGAAPNTVYGLLLCRGDISRsACYDCGTSVWRDAGrAChRAKDVALVYNECYAR
HAP2        LFSTGSNGAAPNTVYGLLLCRGDISRAACYDCGTSVWRDAGSACRRAKDVALVYNECYAR
HAP3        LFSTGSNGAAPNTVYGLLLCRGDISRAACYDCGTSVWRDAGSACRRAKDVALVYNECYAR

HAP1        LSDKDDFLADKVGPGQLTnLMStaNISt-tDiAgYDRAVTqLLAATAEYAAGDIARKLFA
HAP2        LSDKDDFLADKVGPGQLTTLMSSTNISSGADVAAYDRAVTRLLAATAEYAAGDIARKLFA
HAP3        LSDKDDFLADKVGPGQLTTLMSSTNISSGADVAAYDRAVTRLLAATAEYAAGDIARKLFA

HAP1        TGQRVGtDPGFPNLYATAQCAFDITLEACRGCLEGLVARWWDTFPANVaGARmAGPRCLL
HAP2        TGQRVGADPGFPNLYATAQCAFDITLEACRGCLEGLVARWWDTFPANVDGARIAGPRCLL
HAP3        TGQRVGADPGFPNLYATAQCAFDITLEACRGCLEGLVARWWDTFPANVDGARIAGPRCLL

HAP1        RSEVYPFYTGAPMVVLRE
HAP2        RSEVYPFYTGAPMVVLRE
HAP3        RSEVYPFYTGAPMVVLRE
```

Fig. S3. Protein alignment of *OsRLK* from the predicted protein of three haps (HAP1, HAP2 and HAP3) using the EMBL software CLUSTAL 2.0.12 multiple sequence alignment tool. The same color indicates completely conserved amino acid positions.

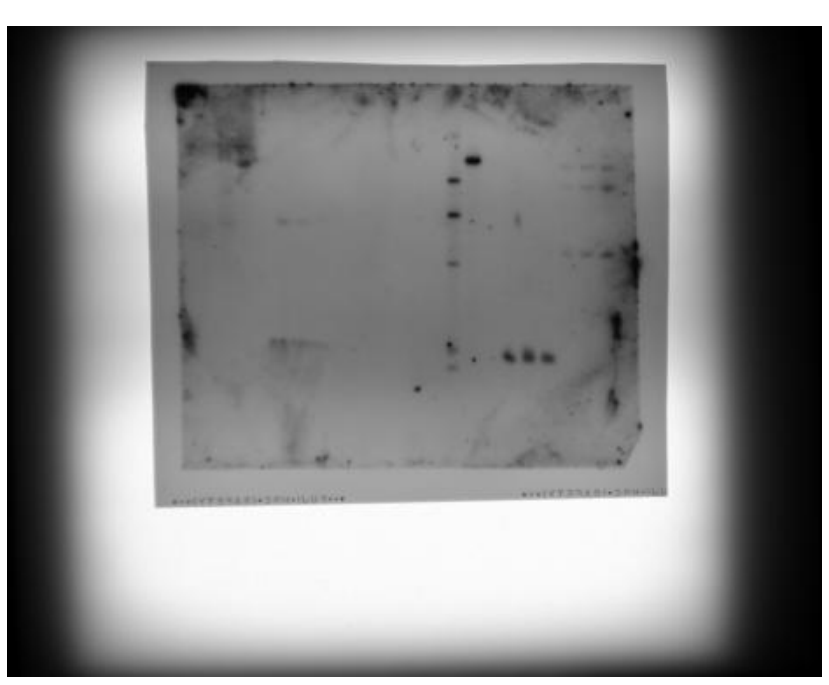

The original gel plot for Fig. S1.
