# Supplementary material for: Wild rice locus enables microbiome re-domestication for enhanced nitrogen-use efficiency
Source: Nat Microbiol. 2026 Sep 30;11(11):3010–24. doi: 10.1038/s41564-026-02498-x (PMC13739750; doi:10.1038/s41564-026-02498-x)
Supplement: Supplementary file 2 [file 41564_2026_2498_MOESM2_ESM.pdf]

nature portfolio

Corresponding author(s): Eiko E. Kuramae
Last updated by author(s): Aug 5, 2026

Reporting Summary

Nature Portfolio wishes to improve the reproducibility of the work that we publish. This form provides structure for consistency and transparency in reporting. For further information on Nature Portfolio policies, see our Editorial Policies and the Editorial Policy Checklist.

Statistics

For all statistical analyses, confirm that the following items are present in the figure legend, table legend, main text, or Methods section.

n/a	Confirmed	
☐	☒	The exact sample size (*n*) for each experimental group/condition, given as a discrete number and unit of measurement
☐	☒	A statement on whether measurements were taken from distinct samples or whether the same sample was measured repeatedly
☐	☒	The statistical test(s) used AND whether they are one- or two-sided<br>*Only common tests should be described solely by name; describe more complex techniques in the Methods section.*
☐	☒	A description of all covariates tested
☐	☒	A description of any assumptions or corrections, such as tests of normality and adjustment for multiple comparisons
☐	☒	A full description of the statistical parameters including central tendency (e.g. means) or other basic estimates (e.g. regression coefficient) AND variation (e.g. standard deviation) or associated estimates of uncertainty (e.g. confidence intervals)
☐	☒	For null hypothesis testing, the test statistic (e.g. *F*, *t*, *r*) with confidence intervals, effect sizes, degrees of freedom and *P* value noted<br>*Give P values as exact values whenever suitable.*
☒	☐	For Bayesian analysis, information on the choice of priors and Markov chain Monte Carlo settings
☒	☐	For hierarchical and complex designs, identification of the appropriate level for tests and full reporting of outcomes
☐	☒	Estimates of effect sizes (e.g. Cohen's *d*, Pearson's *r*), indicating how they were calculated

Our web collection on statistics for biologists contains articles on many of the points above.

Software and code

Policy information about availability of computer code

Data collection	No software was used.
Data analysis	Differences in the rhizosphere bacterial community structure between the parental lines and the two RIL populations were assessed using non-metric multidimensional scaling (NMDS) based on the Bray-Curtis dissimilarity matrix with the vegan package in R (v4.1.2). PERMANOVA was employed to evaluate the statistical significance of clustering patterns observed in the ordination plots. Generalized joint attribute modeling (GJAM) was conducted to determine the core bacteriomes profiles and to identify core bacteriomes of different accessions recruited under similar soil conditions. The gjam package in R (v4.1.2) facilitated the identification of the core rhizobacteriome, consisting of microbes with regression coefficients that were positive and significantly different from zero across the populations. Visualizations, including Sankey diagrams, were created using Origin 2023. Based on the taxonomic classification, a species tree was inferred using GraPhlAn, which displays taxonomic data generated by other metagenomic analysis tools without branch lengths. Correlations among plant physiological and biochemical properties were determined using Pearson's correlation coefficients via the linKEt package in R (v4.1.2). Mantel correlations between core microbial composition and plant physiological and biochemical data, with 9,999 permutations, were analyzed using the vegan package in R (v4.1.2)

For manuscripts utilizing custom algorithms or software that are central to the research but not yet described in published literature, software must be made available to editors and reviewers. We strongly encourage code deposition in a community repository (e.g. GitHub). See the Nature Portfolio guidelines for submitting code & software for further information.

Data

Policy information about availability of data

All manuscripts must include a data availability statement. This statement should provide the following information, where applicable:

- Accession codes, unique identifiers, or web links for publicly available datasets
- A description of any restrictions on data availability
- For clinical datasets or third party data, please ensure that the statement adheres to our policy

The sequences were deposited in the National Center for Biotechnology Information (NCBI; https://www.ncbi.nlm.nih.gov/) under accession number PRJNA1005712. The whole-genome resequencing data of the recombinant inbred line (RIL) populations used for QTL mapping have been deposited in the NCBI SRA under BioProject accession PRJNA1507297. The whole-genome resequencing data of rice accessions 261-382 have been deposited in the NCBI SRA under BioProject accession PRJNA1347954, whereas genome resequencing data of accessions 1-260 (Excel File 4) were obtained from the published dataset PRJEB6180. The RNA-seq data have been deposited in the NCBI SRA under accession PRJNA1508715. The 16S rRNA gene sequences of the nine Acinetobacter strains used in this study have been deposited in GenBank under accession numbers PZ835331–PZ835339. Source data are provided with this paper.

Research involving human participants, their data, or biological material

Policy information about studies with human participants or human data. See also policy information about sex, gender (identity/presentation), and sexual orientation and race, ethnicity and racism.

Reporting on sex and gender	*Use the terms sex (biological attribute) and gender (shaped by social and cultural circumstances) carefully in order to avoid confusing both terms. Indicate if findings apply to only one sex or gender; describe whether sex and gender were considered in study design; whether sex and/or gender was determined based on self-reporting or assigned and methods used. Provide in the source data disaggregated sex and gender data, where this information has been collected, and if consent has been obtained for sharing of individual-level data; provide overall numbers in this Reporting Summary. Please state if this information has not been collected. Report sex- and gender-based analyses where performed, justify reasons for lack of sex- and gender-based analysis.*
Reporting on race, ethnicity, or other socially relevant groupings	*Please specify the socially constructed or socially relevant categorization variable(s) used in your manuscript and explain why they were used. Please note that such variables should not be used as proxies for other socially constructed/relevant variables (for example, race or ethnicity should not be used as a proxy for socioeconomic status). Provide clear definitions of the relevant terms used, how they were provided (by the participants/respondents, the researchers, or third parties), and the method(s) used to classify people into the different categories (e.g. self-report, census or administrative data, social media data, etc.) Please provide details about how you controlled for confounding variables in your analyses.*
Population characteristics	*Describe the covariate-relevant population characteristics of the human research participants (e.g. age, genotypic information, past and current diagnosis and treatment categories). If you filled out the behavioural & social sciences study design questions and have nothing to add here, write "See above."*
Recruitment	*Describe how participants were recruited. Outline any potential self-selection bias or other biases that may be present and how these are likely to impact results.*
Ethics oversight	*Identify the organization(s) that approved the study protocol.*

Note that full information on the approval of the study protocol must also be provided in the manuscript.

Field-specific reporting

Please select the one below that is the best fit for your research. If you are not sure, read the appropriate sections before making your selection.

☐ Life sciences ☐ Behavioural & social sciences ☒ Ecological, evolutionary & environmental sciences

For a reference copy of the document with all sections, see nature.com/documents/nr-reporting-summary-flat.pdf

Ecological, evolutionary & environmental sciences study design

All studies must disclose on these points even when the disclosure is negative.

Study description	We first assessed the rhizosphere microbiota assembly of two Recombinant Inbred Line (RIL) populations representing the progenies of wild rice (O. rufipogon) and cultivated rice (O. sativa L. ssp. japonica and O. sativa L. ssp. indica), and tested whether the paternal wild rice core rhizosphere bacterial community could be reinstated in RILs (each RIL 100 accessions and with 2 replicates) grown under field conditions. We than mapped quantitative trait loci (QTLs) for the abundance of core taxa and overlapped rhizosphere community-associated QTLs with domestication loci identified from whole-genome data of 382 rice accessions (117 wild rice O. rufipogon and 265 cultivated rice O. sativa). Subsequent transcriptome analysis identified genes, particularly the receptor-like kinase-encoding gene OsRLK (Os04g0659300), expressed in these RILs and associated with the abundance of specific bacterial taxa from the core rhizosphere bacterial community. The natural variations in the coding region of OsRLK in the world-wide rice core collection of 105 accessions (including 33 accessions of wild rice and 72 accessions of cultivated rice) were obtained from sequencing. Haplotype

analysis and phylogenetic tree construction were performed based on the sequencing data of 105 germplasm accessions. Lastly, we isolated and constructed an Acinetobacter synthetic community from the transferred core rhizobacteriome to validate the function of Acinetobacter and its association with the OsRLK gene using overexpression and knock-out lines.

Research sample

For the hybridization experiments, we utilized two cultivars of Oryza sativa as female parents: O.sativa L.ssp. japonica (Longgeng 31) and O.sativa L.ssp. indica (Huanghuazhan), paired with O. rufipogen Dongxiang wild rice as the male parent. The resultant F1 hybrids were self-crossed for three generations to generate two distinct F4 RIL populations, designated as RIL-J (based on Longgeng 31) and RIL-I (based on Huanghuazhan). Both RIL populations along with the parental lines, consisting of 100 lines each, were included in this study and were sampling occurred from September 26-30, 2022. To explore rice domestication loci, thousands of wild rice (O.rufipogon) and cultivated rice (O.sativa L.ssp.japonica and O.sativa L.ssp.indica) accessions were planted in the same field next year on May 15, 2023. The field was maintained with a water depth of 10 cm until ten days prior to the full heading. Whole plants, including roots, were harvested between September 20 and 30, 2023. To assess whether the rhizobacteriome of paternal wild rice accessions can enhance plant growth and development, Chinese Dongxiang common wild rice accessions were planted in both sterlized soil and non-sterilized soil. To identify candidate genes regulating the genus Acinetobacter, we planted Chinese Dongxiang common wild rice in two soil conditions: sterilized soil inoculated with Acinetobacter synthetic bacterial communities and sterilized soil only. Root samples were collected 30 days post-transplantation. To test the hypothesis that OsRLK contributes to nitrogen utilization efficiency and influences Acinetobacter, overexpression and knock-out constructs were developed using cultivated rice 1504 and wild rice as the host, respectively. This synthetic microbial communities were inoculated into sterilized soil alongside both overexpressed and wild-type rice material. Each pot received 1.0 ml of Synthetic Communities (with an OD600 of 0.5) mixed with 100 g of black soil, resulting in a final concentration of 106 CFU g-1. Rice plants were grown under controlled conditions: 35 °C with 15 h of light per day and 30 °C during 9 h of darkness, maintaining 70 %–80 % relative humidity. Rhizosphere soil samples and plant samples were collected at 30 days post-inoculation.

Sampling strategy

For the RIL populations, sampling was conducted from September 26-30, 2022. To minimize border effects and the influence of adjacent varieties, plants of the same variety were collected from the central sections of the rice planting areas. For microbiome and plant genome sequencing, six plants from the parental lines and 100 plants from each of the two RIL populations were randomly selected. Two replicates for RIL populations rhizosphere soil, 6 replicates for parental lines and bulk soil. Rhizosphere soil was collected for amplicon sequencing following established protocols. 4 replicates for plant physiological and biochemical indicators. Plant genomic DNA extracted from fresh leaves, with a portion of the leaves utilized for chlorophyll concentration analysis, while the remainder of the plants was processed to determine fresh and dry shoot and root weights, and nitrogen content. Plant RNA extraction was performed on three biological replicates of roots from each treatment using a Promega RNA extraction kit. To determine the copy number of the OsRLK transgene, 10 μg of genomic DNA from both overexpressed and wild-type rice materials. To identify rice gene domestication loci and investigated whether the QTLs related to the transferred core rhizobacteriome overlapped with these loci, we sequenced 382 accessions of both wild and cultivated rice varieties. To minimize border effects and the influence of adjacent varieties, these wild and domesticated rice accessions of the same variety were collected from the central sections of the rice planting areas. Plant genomic DNA was extracted from fresh leaves. To assess whether the rhizosphere microbiome of paternal wild rice accessions enhances plant growth and development, Chinese Dongxiang common wild rice accessions were planted in sterilized soil (W), non-sterilized soil (WN). Rice seeds were surface-sterilized with 2.5 % NaClO and germinated in deionized water for five days at 26 °C in darkness. Seedlings were then transferred to sterilized and non-sterilized black soil, collected from the Northeast Institute of Geography and Agroecology experiment station and sterilized with gamma irradiation at 25 kGy. Rice plants were grown under controlled environmental conditions: 15 h of light at 35°C and 9 h of darkness at 30 °C, with 70 %-80 % relative humidity. Root samples were collected 30 days post-transplantation following previously described methods. Samples were washed with demi water and immediately frozen in liquid nitrogen for RNA extraction using a Promega RNA extraction kit (Promega, China, LS1040). Acinetobacter was isolated from rhizosphere soil samples collected from paternal wild rice. Approximately 0.5 g of rhizosphere soil samples were homogenized in distilled water, and the resultant solution was diluted and cultured on medium at 28 °C for three days. The medium includes potassium nitrate (1.0 g/L), asparagine (1.0 g/L), bromothymol blue (BTB) (0.05 g/L), sodium citrate (8.5 g/L), magnesium sulfate heptahydrate (1.0 g/L), ferric chloride hexahydrate (0.05 g/L), potassium dihydrogen phosphate (1.0 g/L), and calcium chloride dihydrate (0.2 g/L). After four rounds of re-plating on the culture medium plates, individual colonies were selected and identified. To identify candidate genes regulating the genus Acinetobacter, we planted Chinese Dongxiang common wild rice in two soil conditions: sterilized soil inoculated with Acinetobacter synthetic bacterial communities (WA) and sterilized soil (W) only. 1.0 ml of Acinetobacter synthetic bacterial communities (with an OD600 of 0.5) were added to 100 ml of solutions and combined with 100 g of black soil (resulting in a final concentration of 106 CFU g−1) for planting wild rice. Rice seeds were surface-sterilized with 2.5 % NaClO and germinated in deionized water for five days at 26 °C in darkness. Seedlings were then transferred to sterilized and non-sterilized black soil, collected from the Northeast Institute of Geography and Agroecology experiment station and sterilized with gamma irradiation at 25 kGy. Rice plants were grown under controlled environmental conditions: 15 h of light at 35°C and 9 h of darkness at 30 °C, with 70 %-80 % relative humidity. Root samples were collected 30 days post-transplantation. To utilized overexpressed and knock-out rice genotype and synthetic microbial communities to confirm the regulatory relationship between gene OsRLK and Acinetobacter synthetic community. This synthetic microbial communities were inoculated into sterilized soil alongside both overexpressed, knock-out. wild rice and wild-type rice material. Each pot received 1.0 ml of Synthetic Communities (with an OD600 of 0.5) mixed with 100 g of black soil, resulting in a final concentration of 106 CFU g-1. Rice plants were grown under controlled conditions: 35 °C with 15 h of light per day and 30 °C during 9 h of darkness, maintaining 70 %–80 % relative humidity. Rhizosphere soil samples were collected at 30 days post-inoculation (dpi) to measure the Acinetobacter abundance, nifH gene abundance and nitrogenase activity. Rhizosphere soil (0.5 g) was suspended in PBS at a 1:10 (w/v) ratio, vigorously vortexed for 40 s, and then centrifuged at speed for 15 min. The resulting supernatant was subjected to nitrogenase activity quantification using the Soil Nitrogenase (NITS) ELISA Kit (ADANTI, Wuhan, China). At 30 dpi, rice plants were harvested for further analysis, with each treatment including five biological replicates. DNA was extracted from 0.5 g of rhizosphere soil samples collected at 0, 5, 15, and 30 dpi using the Fast DNA SPIN kit (MP Biomedicals, Eschwege, Germany). For evaluated the gain yield of overexpression lines and wild-type, we conducted a field experiment. All transgenic plant materials were planted in Jiangxi Academy of Agricultural Sciences Biological Breeding Base at Yichun (28°14′57″N?115°07′05″E) in 2024 for phenotype investigation. Each line was grown in a two-row plot with 10 plants per row, with a spacing of 16.7 cm between plants and 21.6 cm between rows.

Data collection

For the RIL populations, sampling was conducted from September 26-30, 2022. The loose soil adhering to the roots was shake off by Jingjing Chang, Dianwen Wang, Changji Wang, Yu Sun, Yingnan Pang, Zongmu Yao, Hengfei Zhang, Jianfeng Zhang, Weiqiang Li, Aizhen Liang, Enze Wang, Xiangnan Li, Dazhou Chen, Hongping Chen, Xuewen Chen and Lam-Son Phan Tranand approximately 1 mm of soil attached to the roots was left. We separated the 1 mm of soil from the roots by immersing the root with soil in 5 mL of

sterilized water and vortexed to collect the rhizosphere soil that was tightly attached to root. After a short centrifugation and removal of supernatant, each rhizosphere soil sample was stored at - 80 temperature. We cutted one fresh leaf for extracting genomic DNA and chlorophyll concentration. The rest of the plants were used to determine fresh and dry shoot and root weights. Dianwen Wang and Changji Wang were measured plant height, main spike length, main root length and ground diameter. For the 382 accessions, the whole plants were harvested between September 20 to 30, 2023 by Jingjing Chang, Dianwen Wang, Yingnan Pang, Yuanbo Xie, Weiqiang Li, Jilin Wang and Chunjie Tian. For the pot experiment, root, rhizosphere and plant samples were collected 30 day post-transplantation by Jingjing Chang, Changji Wang, Yu Sun and Yingnan Pang. Acinetobacter was isolated from rhizosphere soil samples collected from paternal wild rice. Approximately 0.5 g of rhizosphere soil samples were homogenized in distilled water, and the resultant solution was diluted and cultured on denitrifying bacteria culture medium at 28 temperature for three days. After four rounds of re-plating on the specific culture medium, individual colonies were selected and identified through 16S rRNA amplicon sequencing. Based on their nitrogen fixation capabilities, nine strains of Acinetobacter were selected. These strains were cultured overnight in tryptic soy broth medium at 37 temperature. Cells from each strain were then washed and combined in equal concentrations to construct a synthetic community of Acinetobacter. Dianwen Wang and Jilin Wang determined the copy number of the OsRLK transgene, 10 μg of genomic DNA from both overexpressed and wild-type rice materials from 30-day-old seedlings was digested with 15 U of HindIII (NEB) for 16 hours. For the yield of overexpression lines and wild-type, at the mature stage, effective panicle number per plant (EPN) and panicle length (PL) were evaluated following the methods described previously (Wang et al 2012). Spikelet number per panicle (SNP), seed setting ratio (SS), grain length (GL), grain width (GW), thousand-grain weight (TGW) and grain weight per plant (GWP) were measured using a yield traits scorer in a high-throughput phenotyping facility (Yang et al 2014) .

Timing and spatial scale

For the RIL populations field experiment, we started on April 10, 2022 and sampled on September 26-30, 2022 which was full heading stage. And for the rice domestication loci, thousands of wild rice and cultivated rice were planted in the same field on May 15, 2023. We sampled on September 20 to 30, 2023 as the same stage as the previous experiment. For the pot experiments, we sampled at 30 days after transplanted the seedlings. For the yield collection, we samples at the end of the rice growth.

Data exclusions

No data were excluded.

Reproducibility

We repeated the pot experiments by using the same methods and materials for more than 4 times and they were successful.

Randomization

For the field experiments,we randomly collected from the central sections of the rice planting areas.

Blinding

The investigators did not implement blinding since our experimental setup involved objective and quantifiable measurements (e.g., plant shoot and root weight, nitrogen content, etc.) minimizing the potential for subjective biases.

Did the study involve field work? ☒ Yes ☐ No

Field work, collection and transport

Field conditions

The experimental plots were established at the demonstration base of the Northeast Institute of Geography and Agroecology, Chinese Academy of Sciences, located at the National Agricultural Station in Liaoheyuan, Jilin Province, China. This site features a typical temperate monsoonal climate and is characterized by black soil as classified by the United States Department of Agriculture (USDA) soil taxonomy. Another plot was Jiangxi Academy of Agricultural Sciences Biological Breeding Base at Yichun (28°14′57″N, 115°07′05″E). The experimental site is located in a typical humid subtropical monsoon region characterized by acid red soils developed into paddy soils under long-term rice cultivation, with high temperature, abundant precipitation (~1600 mm yr^{-1}), and pronounced redox dynamics, providing an ideal environment for studying plant–microbiome interactions and nitrogen cycling processes.

Location

The study area was located in Liaoheyuan, Jilin province, China. For the yield experiment was located in Yichun, Jiangxi province, China.

Access & import/export

No import or export.

Disturbance

Sampling went smoothly without the disturbance.

Reporting for specific materials, systems and methods

We require information from authors about some types of materials, experimental systems and methods used in many studies. Here, indicate whether each material, system or method listed is relevant to your study. If you are not sure if a list item applies to your research, read the appropriate section before selecting a response.

Materials & experimental systems

n/a	Involved in the study
☒	☐ Antibodies
☒	☐ Eukaryotic cell lines
☒	☐ Palaeontology and archaeology
☒	☐ Animals and other organisms
☒	☐ Clinical data
☒	☐ Dual use research of concern
☐	☒ Plants

Methods

n/a	Involved in the study
☒	☐ ChIP-seq
☒	☐ Flow cytometry
☒	☐ MRI-based neuroimaging

Plants

Seed stocks

The wild rice and cultivated rice and RIL populations seeds were given by Jilin Wang and Dianwen Wang.

Novel plant genotypes

Two O. sativa cultivars (japonica Longgeng 31 and indica Huanghuazhan) were female parents, paired with male parent O. rufipogon. F1 hybrids were self-crossed for three generations to generate two F4 RIL populations. OsRLK genomic sequence from Dongxiang wild rice was amplified, cloned into pU1301, sequenced, electroporated into Agrobacterium tumefaciens EHA105, and transformed into rice 1504 via Agrobacterium-mediated method. The OsRLK mutants were generated using CRISPR/Cas9 technology.

Authentication

OsRLK DNA insertions.
